# Somatic mobility of transposons is explosive and shaped by distinct integration biases in *Arabidopsis thaliana*

**DOI:** 10.1186/s13059-026-04149-0

**Published:** 2026-07-04

**Authors:** Heena Ambreen, Basile Leduque, Leandro Quadrana, R. Keith Slotkin, Alexandros Bousios, Hans-Wilhelm Nützmann

**Affiliations:** 1 Department of Biosciences, University of Exeter, Geoffrey Pope Building, Stocker Road, Exeter, EX4 4QD UK; 2 School of Life Sciences, University of Sussex, Brighton, UK; 3 Institute of Plant Sciences Paris‐Saclay (IPS2), Centre National de La Recherche Scientifique (CNRS), Institut National de Recherche Pour L’Agriculture, l’Alimentation Et L’Environnement (INRAE), Université Evry, Université Paris‐Saclay, 91190 Gif Sur Yvette, France; 4 Donald Danforth Plant Science Center, St. Louis, MO USA; 5 Division of Biological Sciences, University of Missouri, Columbia, MO 65211 USA; 6 The Milner Centre for Evolution, Department of Life Sciences, University of Bath, Claverton Down, Bath, BA2 7AY UK

**Keywords:** *Arabidopsis thaliana*, Transposable elements, Somatic transposition, Genomic mosaicism, Integration preferences, Epigenetics, Gene clusters, Resistance genes

## Abstract

**Background:**

The movement of transposable elements (TEs) in somatic cells generates genetic and phenotypic diversity across the soma of individual organisms. This process is especially important in plants that display a sessile lifestyle, can regenerate from somatic cells, and continuously establish their germline from somatic stem cells. Yet, the scale and integration biases by which TEs may shape the plant soma remain unknown.

**Results:**

We adapt high-throughput TE sequencing and introduce a new computational pipeline, “deNOVOEnrich”, to identify and analyse somatic transposition events in *Arabidopsis thaliana*. We detect ~ 200,000 new TE insertions across wild-type and epigenetic mutant backgrounds, revealing en masse active transposition in the plant soma. Similar to germline dynamics, somatic integration is non-random and TE-specific. Focusing on the ONSEN, EVADE and AtCOPIA21 families, we find they preferentially integrate into chromosomal arms and genic DNA, and are enriched over H2A.Z, H3K27me3 and H3K4me1 labelled chromatin. However, ONSEN dynamically targets active chromatin, while EVADE displays a depletion in these regions, consistent with niche targeting at the local epigenetic level and spatial distribution across the genome ecosystem. We further demonstrate that environmentally-responsive genes such as resistance (R) genes and biosynthetic gene clusters are preferred integration targets, suggesting an adaptive potential of somatic transposition.

**Conclusions:**

Our work demonstrates that transposon amplification is an explosive and structured event that may contribute to the generation of cellular mosaicism in the soma of *A. thaliana*. We speculate that this phenomenon is common across plants under permissive conditions, both in experimental and natural settings.

**Supplementary Information:**

The online version contains supplementary material available at https://doi.org/10.1186/s13059-026-04149-0.

## Background

Transposable elements (TEs) typically occupy large fractions of eukaryotic genomes, and are crucial for generating novel genetic and phenotypic variation [1–3]. The major contribution of TEs in shaping genome structure and function is well-documented across host taxa [4, 5], however, much of our understanding of the genome-wide impact of TEs has been shaped by studies focussing on heritable transposition events. In contrast, research on the large-scale dynamics of somatic TEs has not been on par with exploring insertions of TEs in the host germline [5], particularly in plant species. One possible explanation is the long-held idea of complete suppression of TE activity in somatic cells [6, 7]. Furthermore, new transposon insertions in the soma are typically not inherited through generations, and are thus considered to have limited evolutionary relevance for both the host and the TE [7, 8]. In addition, somatic TE insertions are rare variants limited to single cells or small cell populations, and, hence, their detection is technically daunting and a plausible reason for underestimating levels of somatic TE transposition [9, 10].

Several studies have provided evidence for frequent TE movement in somatic cells [10–12]. In humans and mice, LINE-1 elements transpose extensively in brain cells, introducing non-heritable changes in neuronal somatic genomes that contribute to cellular heterogeneity and may potentially influence cognitive functions [11, 13]. Similar release of high transposition activity has been observed in cancerous and aged somatic cells in humans [14–16]. In *Drosophila melanogaster*, regular TE movement has been characterized in brain and gut tissues, as well as in aged individuals, demonstrating that somatic mosaicism mediated by TE insertional mutagenesis is an ongoing genomic process [10, 17, 18]. Outside these model metazoans, TE mobilization in somatic tissues is poorly characterised at genome-wide scales.

In plants, the implications of somatic transposition are more far-reaching than in animals. Unlike animals, plant cell differentiation is not dependent solely on cell division lineage patterns, and cells are not set aside early in development for the germline; instead, plants establish their germline in late adulthood from stem cells. In addition, upon wounding or induced in the laboratory, any somatic plant cell has the ability to redifferentiate and contribute to the production of germ cells. This suggests that TE insertions in somatic genomes have higher likelihood of transmitting into the progeny and become a source of novel genetic variability [5]. Yet, most studies have focussed on forward genetics approaches to explain phenotypes showing somatic variegation caused by random TE insertions [19–23] or detected clonal diversity in vegetatively propagated crop species that is derived from TE transposition [24–28].

In maize, studies have shown that somatic transposition leads to differentially colored sectors in kernels [29, 30], while stress-induced transcriptional activation of TEs in somatic cells is well documented in plants [31, 32]. These studies are consistent with TEs being active in the plant soma and important for phenotypic somatic diversity and plant stress physiology. However, the large-scale dynamics of somatic transposition are poorly characterised in plants, leaving a significant gap in our understanding of genome-wide rates of somatic TE proliferation and their impact on genome stability, function and evolution. In addition, investigating somatic transposition in-depth provides a unique opportunity to capture de novo integration patterns of TEs before selection removes deleterious insertions [33, 34], which is a fundamental aspect of TE and genome biology.

Here, using the well-characterized TE mobilome in *Arabidopsis thaliana*, we set out to reliably assess the large-scale dynamics of somatic transposition in a plant species, and to determine the de novo integration preferences of actively transposing TEs. To release TE suppression and trigger high TE activity in our model system, we employ heat stress and epigenetic mutants. We then apply transposable element display sequencing (TEd-seq) [35] to identify rare TE-associated fragments from targeted genomic libraries, and design a new computational pipeline, “deNOVOEnrich” to detect and characterize somatic TE insertions to base pair resolution. This set-up enables us to identify over 200,000 new transposition events for three exemplar LTR retrotransposon families. Analysis of their integration sites reveals shared and unique genomic and epigenomic target site preferences, and a distinct preference for environmentally-responsive genes such as resistance (R) genes and biosynthetic gene clusters. Overall, we provide evidence that TEs mobilize en masse in somatic cells of *A. thaliana* plants, opening the field for future studies on the functional impact of somatic genome plasticity across species.

## Results

### Monitoring active TEs in *Arabidopsis* seedlings

An essential requirement to study somatic insertions is the identification of TE families that can retain high levels of activity and generate large numbers of integration events. To boost transposition in *A. thaliana*, we used four epigenetic mutant lines that are known to compromise TE silencing, and exhibit elevated levels of TE transcription i) the methyltransferase 1 (*met1*) mutant that leads to loss of maintenance of cytosine methylation in the CG sequence context [36], ii) the plant-specific RNA polymerase IV (*polIV*) mutant that is crucial for small interfering RNA (siRNA) production during the upstream phase of RNA-directed DNA methylation (RdDM) [37], iii) the plant-specific RNA polymerase V (*polV*) mutant that produces the scaffold transcript of the TE to anchor the RdDM machinery to chromatin [38, 39] and iv) the *polIVpolV* double mutant (Additional file 1: Table S1). We examined several TE families that are known to be “epigenetically-induced” in these *A. thaliana* mutants [40–42]: the LTR retrotransposon families EVADE (AtCOPIA93), AtCOPIA21, AtCOPIA63, AtCOPIA51, AtCOPIA31, ATGP3 and AtCOPIA52 (SISYPHUS). We also applied heat stress to our plants, because of the presence of heat-responsive TEs, namely the LTR retrotransposon ONSEN (AtCOPIA78) family that has been thoroughly studied as a model TE for stress-mediated activation in plants [31, 43, 44]. Our set of TE families contain between one and eleven intact elements in the *A. thaliana* Col-CEN reference genome and overall occupy 0.16% of genomic DNA (Additional file 1: Table S2).

**Fig. 1:**
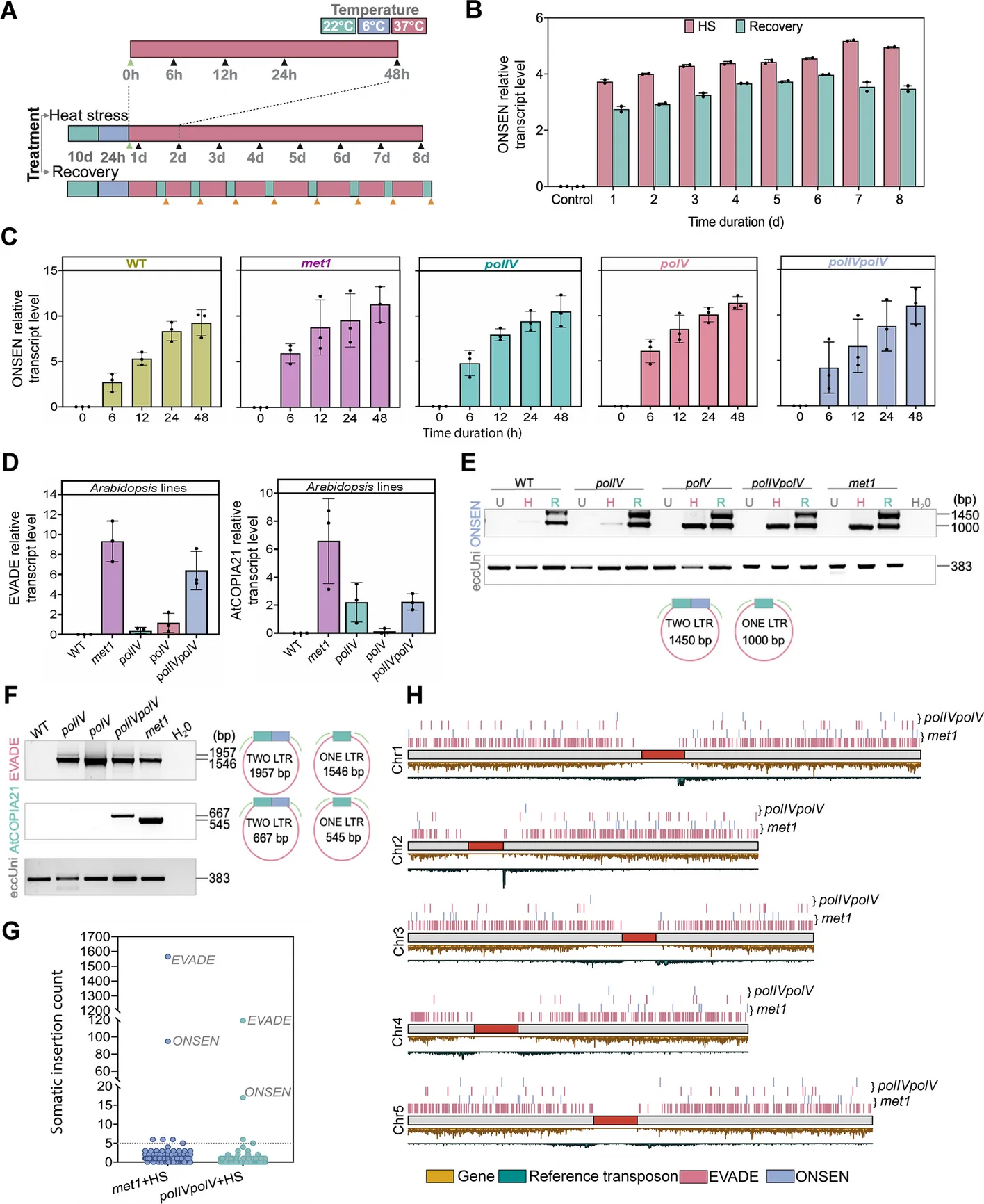
Activation of TE families in *A. thaliana* seedlings.** A** Heat treatments and timepoints for assessing ONSEN transcription levels with qRT-PCR. Sampling timepoints are indicated with green (control), black (heat stress), and orange (recovery) arrowheads. Recovery samples were taken after incubation for 48 h at 22 °C post the heat stress. **B** ONSEN qRT-PCR expression profiles under 8-days heat regime (*n* = 2). Leaf tissues from ~ 100 seedlings were pooled for each replicate. **C** ONSEN qRT-PCR expression profiles at regular intervals extending up to 48 h in wild-type (WT) and mutants (*n* = 3). Expression (log-transformed) was normalized to the unstressed tissue of the corresponding genotype to study induction kinetics within each genotype. The cross-genotype comparison of ONSEN induction at 48 h of heat stress is shown in Additional file 2: Fig. S3A. **D** Relative expression levels of EVADE and AtCOPIA21 (*n* = 3) in unstressed tissue of *Arabidopsis* lines. Mean ± SD plotted for qRT-PCR analysis in **B**-**D**. **E**,** F** Inverse PCR profiles for detection of eccDNA for ONSEN (**E**) EVADE and AtCOPIA21 (**F**). Unstressed (U), 48 h heat-stressed (H) and 48 h recovery post HS (R) samples were used for ONSEN. Unstressed plant material was used for EVADE and AtCOPIA21. Expected size for two and single LTR products have been indicated. eccDNA molecule (eccUni) universally observed in *Arabidopsis* tissues [46] has been used as positive control. Note that additional AtCOPIA21 eccDNA forms are shown in Additional file 2: Fig. S3B. Uncropped gel images are provided in Additional file 3. **G** Number of de novo somatic insertions of different TE families identified in *met1* and *polIVpolV* heat-stressed leaf tissues using whole genome re-sequencing. **H** Genome-wide distribution of somatic ONSEN and EVADE insertions shown in (**G**). Gene and reference TE densities are shown in 10 kbp windows. Centromere location has been shown by red blocks

Our current understanding of ONSEN heat-induction is limited to an application period of 24–48 h [31, 43, 45]. To better discern when maximal transcription is achieved, we profiled ONSEN transcript levels with qRT-PCR in a wider range of heat exposure and treatments (Fig. 1A-C; Additional file 2: Fig. S1; see Methods). In leaf tissues of wild-type plants, ONSEN transcription showed a continuous increase for seven days of heat exposure (37 °C), beyond which transcript levels fell and seedlings died (Fig. 1A, B; Additional file 2: Fig. S2A). Cold priming did not yield any significant effect on transcript abundance (Additional file 2: Fig. S1), while sampling after a 48-h recovery phase decreased ONSEN transcript levels in relation to sampling directly after the heat-stress (Fig. 1B). The four mutant lines followed similar heat-responsive kinetics as wild-type plants, whereby ONSEN transcription correlated strongly with the duration of applied heat (Fig. 1A, C). However, cross-genotype comparison of ONSEN transcript levels at 48 h HS revealed higher induction in RdDM mutants than in *met1* and WT seedlings, consistent with findings of higher ONSEN accumulation in mutants impaired in siRNA biogenesis by Ito et al. [31] (Additional file 2: Fig. S3A). Further, all lines showed adverse growth damage after three days, with symptoms being most severe in *polIVpolV* mutant plants (Additional file 2: Fig. S2B, C). Among the other TE families, we could detect transcripts based on the qRT-PCR profiling only for EVADE and AtCOPIA21 in the epigenetic mutant lines, with *met1* showing the highest activation (Fig. 1D).

As a more direct marker of TE mobility, we detected TE extrachromosomal circular DNA (eccDNA) that is generated after reverse transcription [47]. We examined the formation of eccDNA by inverse PCR profiling that captures LTR junctions of eccDNA molecules. Our analysis of seedlings exposed to a 48-h heat-stress with and without a 48-h recovery phase revealed the presence of two abundant forms of ONSEN eccDNA with one or two LTRs in all lines (Fig. 1E). The two-LTR eccDNA product was prominent in the recovery phase, consistent with extrachromosomal DNA copies accumulating in the cytoplasm after the synthesis of excessive levels of TE transcripts, or with integration of new copies slowing down after the heat-stress window. EVADE eccDNA was abundant across all mutant lines and mostly contained one LTR (Fig. 1F). AtCOPIA21 eccDNA was detected only in the *met1* and *polIVpolV* double mutant wherein both LTR forms were present (Fig. 1F; Additional file 2: Fig. S3B). Identity of the variable LTR forms of ONSEN and EVADE eccDNA was confirmed by Sanger sequencing (Additional file 2: Fig. S4). We also found varying levels of eccDNA for more families in our mutant lines but not the wild-type controls (Additional file 2: Fig. S5), indicating distinct interactions between TEs and the epigenetic silencing pathways. Collectively, our assessment of known environmentally and epigenetically-induced TE families using qRT-PCR and eccDNA analyses points towards broad activation of TEs, with ONSEN, EVADE and AtCOPIA21 showing the highest levels of activity.

### Whole genome sequencing supports somatic transposition of ONSEN and EVADE

To explore whether high levels of transcription and eccDNA can lead to transposition in somatic cells, we performed high coverage (> 100 × fold) Illumina short-read whole-genome sequencing on 48 h heat-stressed leaf tissues pooled from ~ 100 seedlings of *met1* and *polIVpolV,* the two mutant backgrounds where most TE families show evidence of high activity (Fig. 1A-F, Additional file 2: Fig. S5). We mapped de novo somatic insertions with the TEMP2 software [48], which uses split and discordant reads for the identification of novel insertions. Each identified insertion was allocated to a specific family using the curated TE library of the Col-CEN reference genome. We detected 1,803 and 198 putative insertions in *met1* and *polIVpolV*, respectively, with seven families substantially contributing to transposition load by having ≥ 5 insertions (Fig. 1G) (Additional file 4). ONSEN and EVADE exhibited the highest levels of mobility with 112 (5.6%) and 1,684 (84.2%) insertions for both mutants combined (Fig. 1G). The majority of these insertions were localized on the chromosomal arms (Fig. 1H).

### Explosive bursts of somatic transposition detected by targeted sequencing

**Fig. 2:**
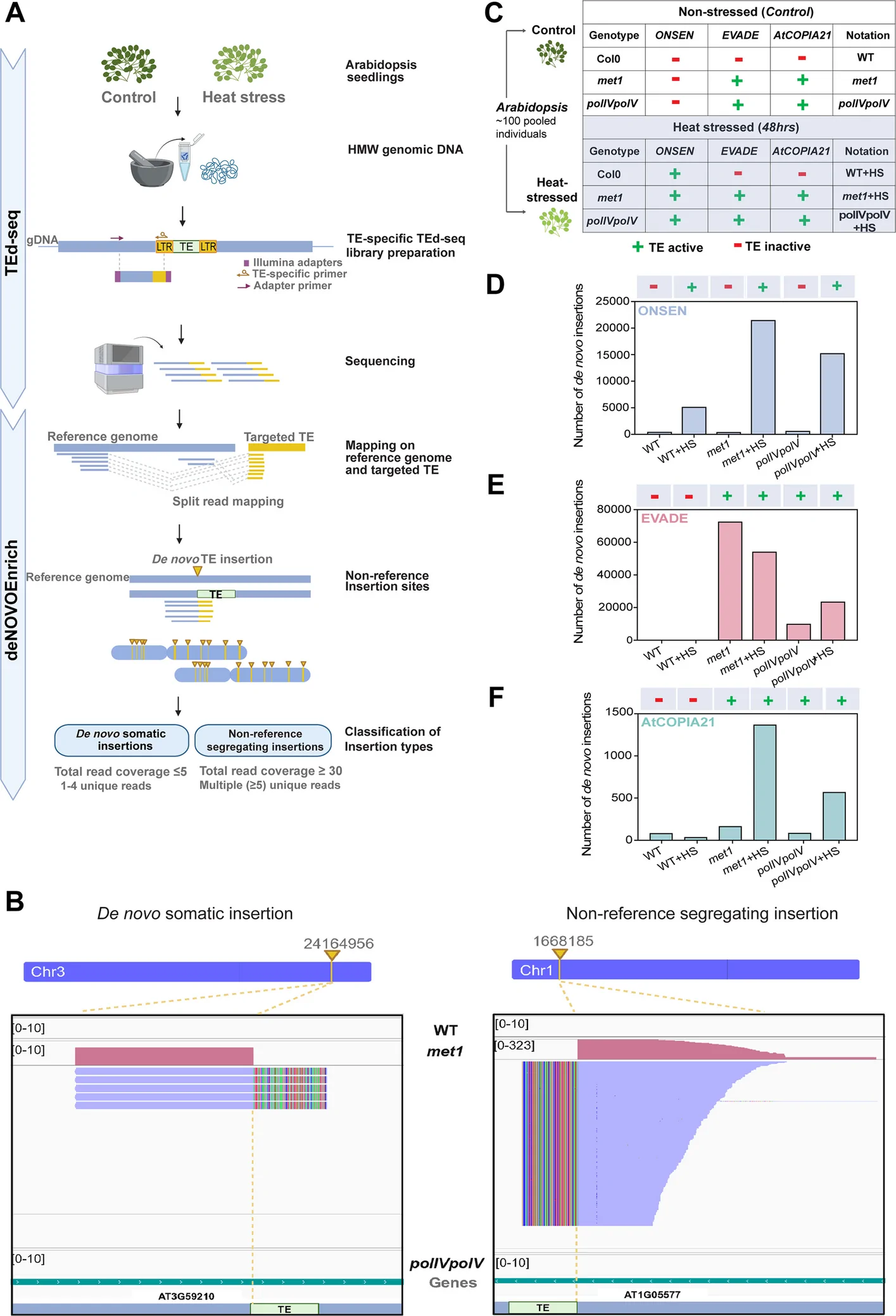
Somatic transposition of ONSEN, EVADE and AtCOPIA21 in *A. thaliana* leaves. **A** Workflow for the precise detection of de novo somatic and non-reference segregating insertions. TEd-seq: Targeted sequencing libraries are generated for specific TEs and sequenced at high coverage with Illumina. deNOVOEnrich*:* New insertion sites are identified based on split-reads, whereby reads that partially map to the reference genome, and their unmapped region shows strong homology to the TE end, are used to identify genome-wide transposition events. The insertions are classified as somatic or non-reference segregating insertions based on coverage thresholds (see “Methods” and Additional file 2: Fig. S6 for step-by-step details on the deNOVOEnrich pipeline). **B** Genome browser view of representative de novo somatic (left) and non-reference segregating (right) insertions of EVADE in the *met1* mutant. Read coverage (pink) and alignment (blue) tracks are shown for the TE-genome junction of the new insertions. Clipped read segments corresponding to the TE end are indicated by rainbow-colored portions. Tracks for the same genomic location in wild-type and *polIVpolV* are included. Chromosomal position (top) and the specific insertion coordinates within two different genes (bottom) are shown in the ideograms. (for information on non-reference segregating insertions identified in our analysis see Additional file 2: Supplementary Information 1 and Fig. S20). **C** Experimental conditions used for TEd-seq. The expected activity status (“ + ” for active, “-” for inactive) is shown for each combination of genome background and TE family. **D**-**F** Number of somatic TE insertions for ONSEN (**D**), EVADE (**E**) and AtCOPIA21 (**F**) in experimental combinations of (**C**). The expected activity status is indicated above each plot. Figure 2A created in Biorender

While high coverage whole-genome sequencing can provide evidence for somatic TE mobilization, the inherent limited sensitivity of a genome-wide approach restricts our ability to assess the true extent of integration in the somatic pool of cells. We therefore leveraged a recently developed TE-enrichment sequencing approach, TEd-seq, that is designed to identify non-reference low frequency insertions for single TE families with ultra sensitivity and specificity in large numbers of individuals, such as in “evolve and resequence” population experiments [35, 49] (Fig. 2A). To adapt the bioinformatic analysis to the expected lower frequency of TE insertions within a DNA sample of somatic tissue, we developed a new computational pipeline, termed “deNOVOEnrich” [50]. This pipeline uses deeply-filtered, high-confidence split reads to map the de novo insertional landscape to base pair resolution (Fig. 2A, Additional file 2: Fig. S6) (see “Methods”). deNOVOEnrich applies read coverage to differentiate single-generation somatic (≤ 5 reads) from non-reference segregating insertions (≥ 30), with the latter occurring, for example, during seed propagation cycles prior to TEd-seq application (Fig. 2A, B; Additional file 2: Fig. S6).

Based on our initial screenings for active TE families (Fig. 1), we generated TEd-seq data for ONSEN, EVADE and AtCOPIA21 in wild-type, *met1* and *polIVpolV* mutant plants under stress (37 °C for 48 h) and non-stress conditions (Fig. 2C) [51]. These combinations generate predictions about the activation patterns of each family, which then serve as controls to estimate false discovery rates (FDR) and enhance the robustness of the analysis. For example, ONSEN is not anticipated to transpose in the absence of heat-stress in either wild-type or mutant plants, EVADE should only transpose in mutant lines regardless of the application of heat, and no family should transpose in wild-type with no heat-stress (Fig. 2C). Given the absence of known activation stimuli in control samples, any insertion events detected in these controls likely represent background noise or technical artifacts and thus are considered false positives. Across all TEd-seq libraries, we generated 132.5 Gbp of data using 2 × 150 bp paired-end reads, which ranged between 8.8 to 32.6 Gbp per library and was derived from leaves of a pool of ~ 100 seedlings (Additional file 1: Table S3).

After normalising for library size, we detected for ONSEN a total of 5,168, 15,274 and 21,509 somatic insertions in heat-stressed wild-type, *polIVpolV* and *met1* plants compared to 459, 633 and 421 in non-stress conditions of the same lines (Fig. 2D, Additional file 2: Fig. S7). This represents a 11-, 24- and 51-fold increase and is consistent with low FDRs in our pipeline*.* Notably, ONSEN showed significantly higher levels of transposition in the mutant lines, indicating enhanced propagation in epigenetically repressed conditions in addition to the effect of heat. For EVADE, loss of epigenetic control resulted in massive propagation in somatic cells of non-stressed *polIVpolV* and *met1* plants with 10,020 and 72,679 new insertions respectively, which are 51 and 367 times higher than the FDR levels observed in non-stressed wild-type (198 insertions) (Fig. 2E, Additional file 2: Fig. S8). Application of heat in the mutant plants did not significantly alter transposition rates (1.3–twofold difference; 23,623 insertions in *polIVpolV* and 54,223 in *met1*) (Fig. 2E, Additional file 2: Fig. S8). In contrast to ONSEN and EVADE, AtCOPIA21 displayed less intense transposition in the mutant lines (Fig. 2F, Additional file 2: Fig. S9). In fact, the most notable increase was observed in the heat-stressed mutant plants, which showed > 5 fold difference in relation to wild-type (571 and 1,371 in *polIVpolV* and *met1*, respectively). Taken together, our TEd-seq/deNOVOEnrich analysis has captured hundreds of thousands of new insertions in the Arabidopsis soma, establishing that somatic transposition is an explosive ongoing event under conditions of weakened epigenetic control or environmental stress.

### Genomic features influencing de novo somatic integration

Analysis of TE insertion patterns often show non-random distribution along chromosomes, which can be attributed to the combined effect of integration preferences and post-integration selection acting on each site [34, 52]. In plants, TE integration patterns have been mostly studied by analysing chromosomal distribution of TEs in generations subsequent of TE amplification (i.e. post-selection), which obscures true insertion biases. Our investigation, however, of genome-wide de novo somatic transposition within a single generation provides a unique opportunity to capture integration patterns before selection alters their distribution.

**Fig. 3:**
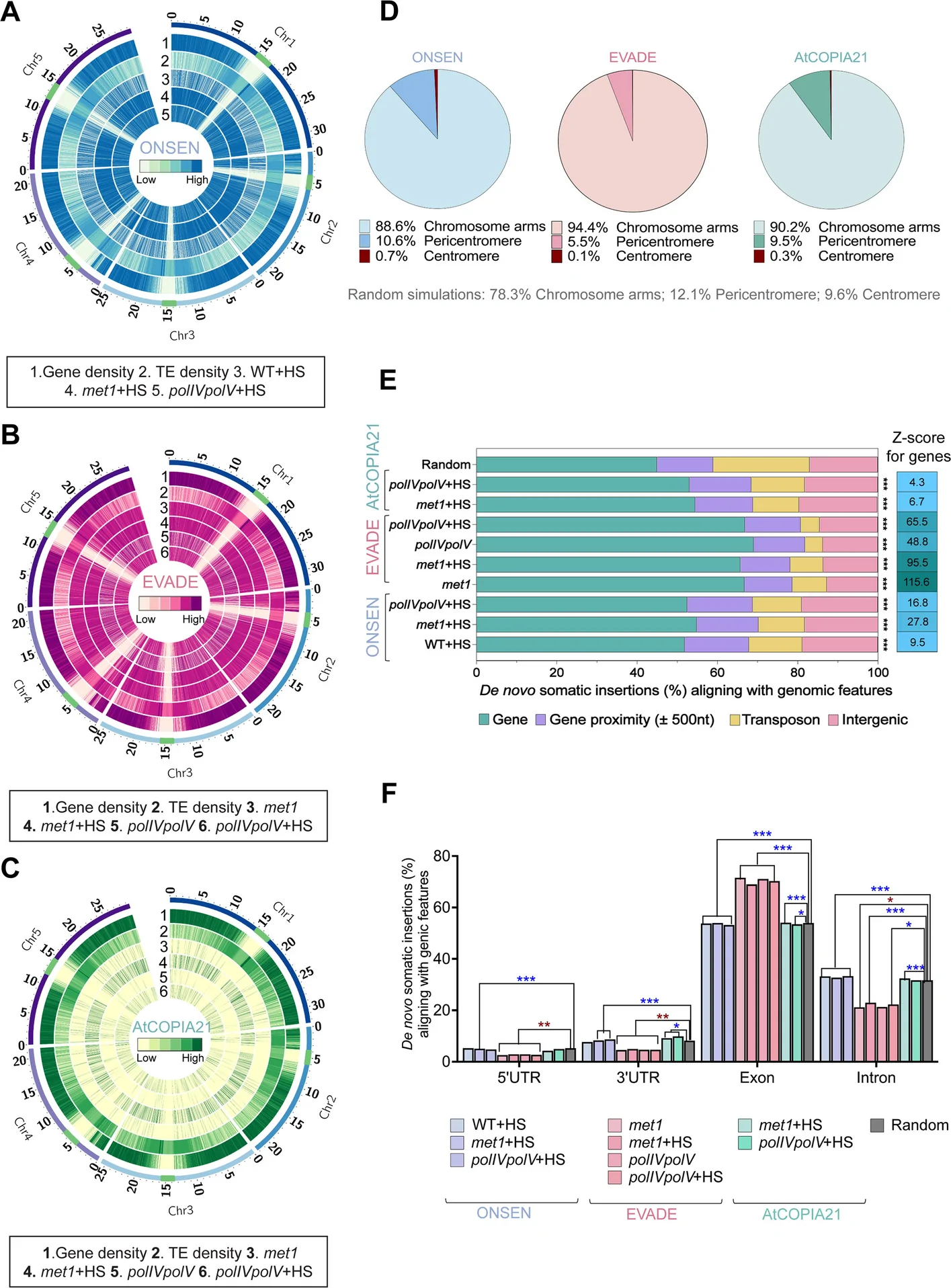
Topology of somatic insertions in the *A. thaliana* genome. **A-C** Circos plots showing somatic insertions along the *A. thaliana* chromosomes for ONSEN (**A**), EVADE (**B**), and AtCOPIA21 (**C**). Green blocks in outermost ribbons indicate centromere positions. Gene and TE density have been calculated in 20 kb non-overlapping windows. **D** Proportion of somatic insertions in chromosomal arms, pericentromeres and centromeres. To calculate the expected frequency of insertion in the three main genomic compartments, simulations were conducted 1000 times with permutation test. Z scores are shown in Additional file 2: Fig. S10A. **E** Fraction of somatic insertions in genes, 500 bp windows proximal to genes, reference TEs and intergenic regions. Permutation tests were applied to evaluate the level of significance for each combination of genome background and TE family (***p < 0.001; Z scores are shown for genes). Z scores for other genomic regions are provided in Additional file 2: Fig. S10B. For AtCOPIA21, only heat-stressed samples of mutant lines were analysed, following the number of somatic insertions detected as shown in Fig. 2F. **F** Fraction of somatic insertions in different genic components. Permutation tests were applied to evaluate the level of significance for genic components and somatic insertions (***p < 0.001, **p < 0.01, *p < 0.05). Positive association marked by blue asterisk while negative association shown by red asterisk. Only statistically significant relationships have been indicated. Insertion density per Kb across genic components and permutation Z scores are provided in Additional file 2: Fig. S11

We first explored the distribution of somatic insertions in the three main compartments of the *A. thaliana* genome, the centromeres (9.6% of genomic DNA), pericentromeres (12.1%), and chromosomal arms (78.3%). Centromere boundaries were defined in the Col-CEN reference assembly [53], while the junctions between the pericentromeres and the chromosomal arms were recently resolved based on mapping thousands of meiotic recombination crossovers [54]. Reflecting a strong affinity for euchromatin, all three families, and especially EVADE (94.4% of insertions), showed a universal preference to integrate into chromosome arms (Permutation test; *p* < 0.001) (Fig. 3A-D; Additional file 2: Fig. S10A), while being underrepresented in pericentromeres and absent from centromeres that are only invaded by ATHILA LTR retrotransposons [53, 55] (Fig. 3A-D; Additional file 2: Fig. S10A).

We further partitioned the genome into (i) genes, (ii) 500 bp regions flanking genes, (iii) reference TEs, and (iv) intergenic space to associate somatic integration with underlying genomic features at the regional scale. Among the three families, and following the strong bias for euchromatin, EVADE was significantly enriched in genes (67% on average across all mutants, Permutation test; *p* < 0.001) (Fig. 3E; Additional file 2: Fig. S10B), and, in particular, exonic DNA (Permutation test; *p* < 0.001) (Fig. 3F; Additional file 2: Fig. S11), while being substantially less present within the TE space (Fig. 3E; Additional file 2: Fig. S10B). Further zooming in the nucleotide composition around the insertion sites did not reveal any consensus motif (Additional file 2: Fig. S12). ONSEN and AtCOPIA21 also generated statistically significant enrichment for euchromatin and genes in most cases, albeit not as noticeable as EVADE (Fig. 3D, E; Additional file 2: Figs. S10-S11).

### Epigenomic signatures define somatic integration bias of TE families

**Fig. 4:**
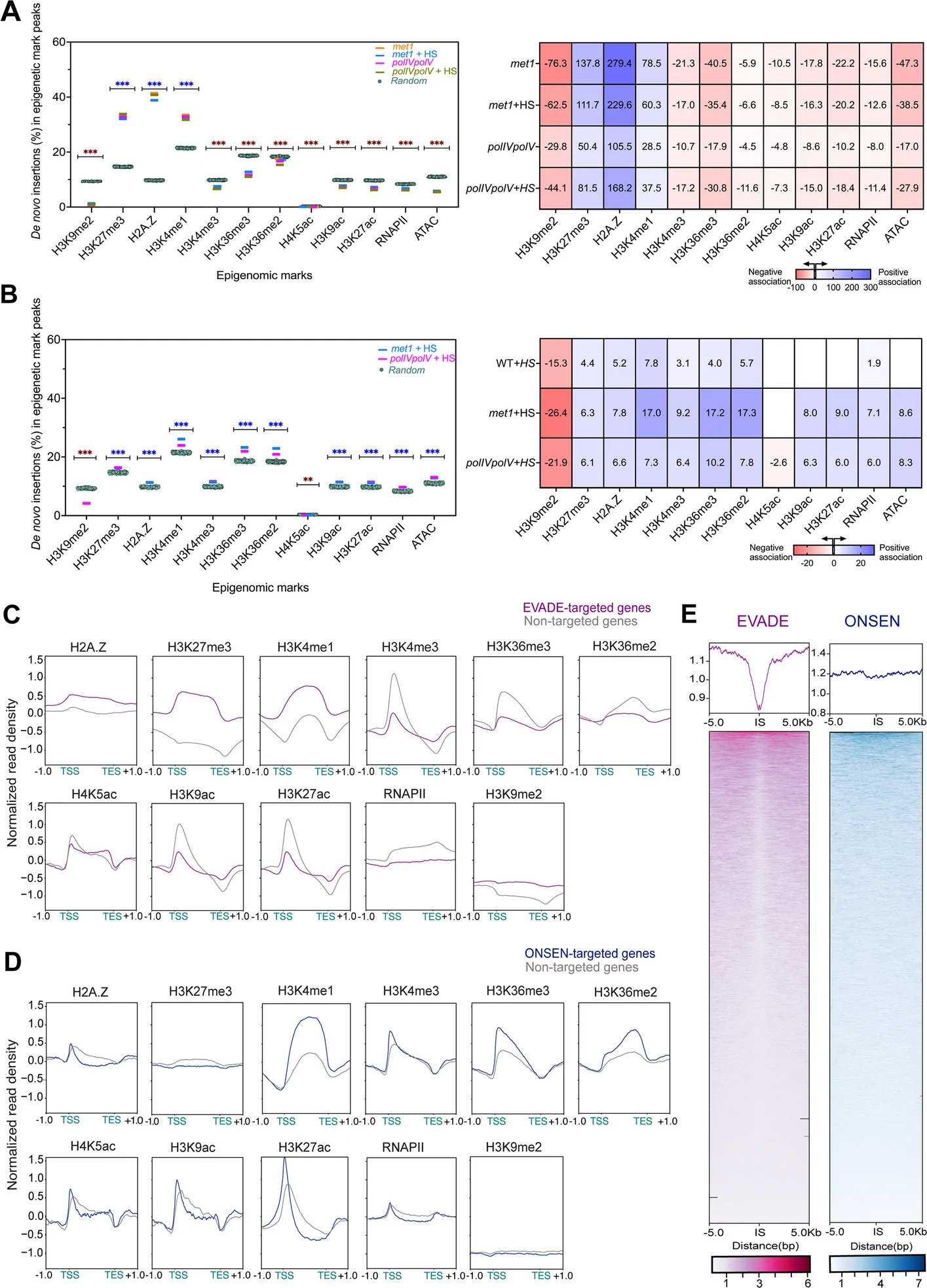
Epigenomic landscape of somatic insertion sites (**A**, **B**). Proportional distribution of de novo somatic insertions of EVADE (**A**) and ONSEN (**B**) in the peak regions of various epigenomic marks and chromatin accessibility, ATACseq (left). Coloured horizontal lines represent observed values, while teal-green dots indicate expected values for the randomized insertions. Permutation test was applied to evaluate level of significance (***p < 0.001; **p < 0.01). Positive association marked by blue asterisk while negative association shown by red asterisk. Heatmap depicting Z-score representing the strength of association between somatic insertions of EVADE and ONSEN, and epigenomic marks, computed through permutation tests (right). Positive association shown by blue gradient while negative association represented by red gradient. Empty blocks show samples with non-significant associations. Insertion density per Kb across epigenomic features is presented in Additional file 2: Fig. S14. **C**, **D** Metagene plots illustrating the levels of various epigenetic marks over genes targeted for de novo somatic insertions by EVADE (**C**) and ONSEN (**D**) compared to a randomized set of genes with no insertions from the respective TE family. **E** Metagene and heatmap profiles at and around (± 5 kb flanking region) the insertion sites of EVADE and ONSEN integration events for *met1* + HS samples generated using ATACseq data. Metagene profiles for other samples are shown in Additional file 2: Fig. S16. Metagene plots in C-E are generated using datasets from *met1* mutant and WT unless otherwise stated. Dataset IDs are shown in Additional file 1: Table S4

Integration preferences for some transposons have been shown to be influenced by the epigenomic state of the underlying chromatin [34, 41, 49]. We therefore investigated if the sites of de novo somatic insertions are correlated with specific chromatin features including histone variants and modifications (source datasets provided in Additional file 1: Table S4). Aligning with the observed distribution confined to the chromosomal arms, somatic insertions from all the TE families showed a significant depletion in genomic regions marked by the constitutive heterochromatin mark, H3K9me2 (Fig. 4A, B; Additional file 2: Figs. S13-S14). De novo EVADE somatic insertions were enriched in genomic regions characterised by repressive facultative heterochromatin mark H3K27me3, histone variant H2A.Z that is associated with both gene activation and repression, and histone mark H3K4me1, which is a distinctive signature for active genes (Fig. 4A; Additional file 2: Fig. S14). Remarkably, all other investigated active chromatin marks were significantly depleted in regions of EVADE somatic insertions (Fig. 4A; Additional file 2: Fig. S14). To substantiate our findings, we assembled 2,472 genes that are commonly targeted by new EVADE somatic integrations in all samples and evaluated the type and extent of chromatin signatures over their body compared to a random set of genes without EVADE insertions. We observed high signal deposition of H3K27me3, H2A.Z, and H3K4me1 over the gene bodies of EVADE-targeted genes while a contrasting trend was observed for the other active marks (Fig. 4C). Consistent with this, clustering of EVADE-targeted genes based on epigenomic signatures showed a strong enrichment for all three marks individually and in combination (Additional file 2: Fig. S15A, B).

Like EVADE, the somatic insertions of ONSEN showed significant integration preferences for H3K27me3, H2A.Z, and H3K4me1, yet with lower strength of association (Fig. 4B; Additional file 2: Fig. S14). In contrast to EVADE, we also observed a substantial enrichment of regions occupied with RNAPII and other active chromatin marks including H3K4me3, H3K36me3, H3K36me2, H3K27ac and H3K9ac in the mutant lines (Fig. 4B; Additional file 2: Fig. S14). Notably, ONSEN integrations in wild type *Arabidopsis* did not exhibit a target preference towards two of the additional active marks, H3K27ac and H3K9ac (Additional file 2: Figs. S13A, S14). The quantification of chromatin marks over 449 genes targeted by ONSEN somatic insertions across all samples by and large mirrored the genome-wide patterns. However, no obvious enrichment for H2A.Z and H3K27me3 was detected, suggesting that insertional preference of ONSEN for H2A.Z and H3K27me3 is not limited to genic targets (Fig. 4D). Clustering based on epigenomic profiling highlighted that the majority of these genes are characterized by a predominant co-occurrence of H2A.Z, H3K4me1 and other active chromatin signatures (Additional file 2: Fig. S15C, D). To verify the contrasting integration choices of EVADE and ONSEN for active chromatin marks, we further analysed the chromatin landscape at and around the integration sites using ATACseq data. EVADE integration sites were depleted in open chromatin regions, while ONSEN integration sites showed a mild tendency for open accessible chromatin (Fig. 4E; Additional file 2: Fig. S16). Next, we evaluated the TE insertion preferences in contrasting DNA methylation features that uniquely characterize *Arabidopsis* genotypes – gene body methylation in WT and hypo-differentially methylated regions (hypoDMRs) in *met1*. We observed that ONSEN insertions in WT were enriched in gene body methylated genes (permutation test; *p*-value < 0.01) in line with their insertional preference for active chromatin (Additional file 2: Fig. S17A). In contrast, EVADE insertions were depleted from hypoDMRs in *met1* and *met1* + HS (*p*-value < 0.001; Additional file 2: Fig. S17B). ONSEN insertions showed no significant association with hypoDMRs in *met1* (*p*-value > 0.05). Finally, the overall integration trends of AtCOPIA21 closely resembled those of ONSEN suggesting an insertional preference for active regions. However, these associations were not significantly enriched across all active marks and samples (Additional file 2: Fig. S13B,C; Figs. S14, S17B). Together, our findings provide evidence that real-time somatic transposition in *A. thaliana* presents family-specific biases towards distinct chromatin environments, expanding the spectrum of chromatin features that guide TE integration patterns.

### Insertion hotspots characterized by recurrent somatic insertions

**Fig. 5:**
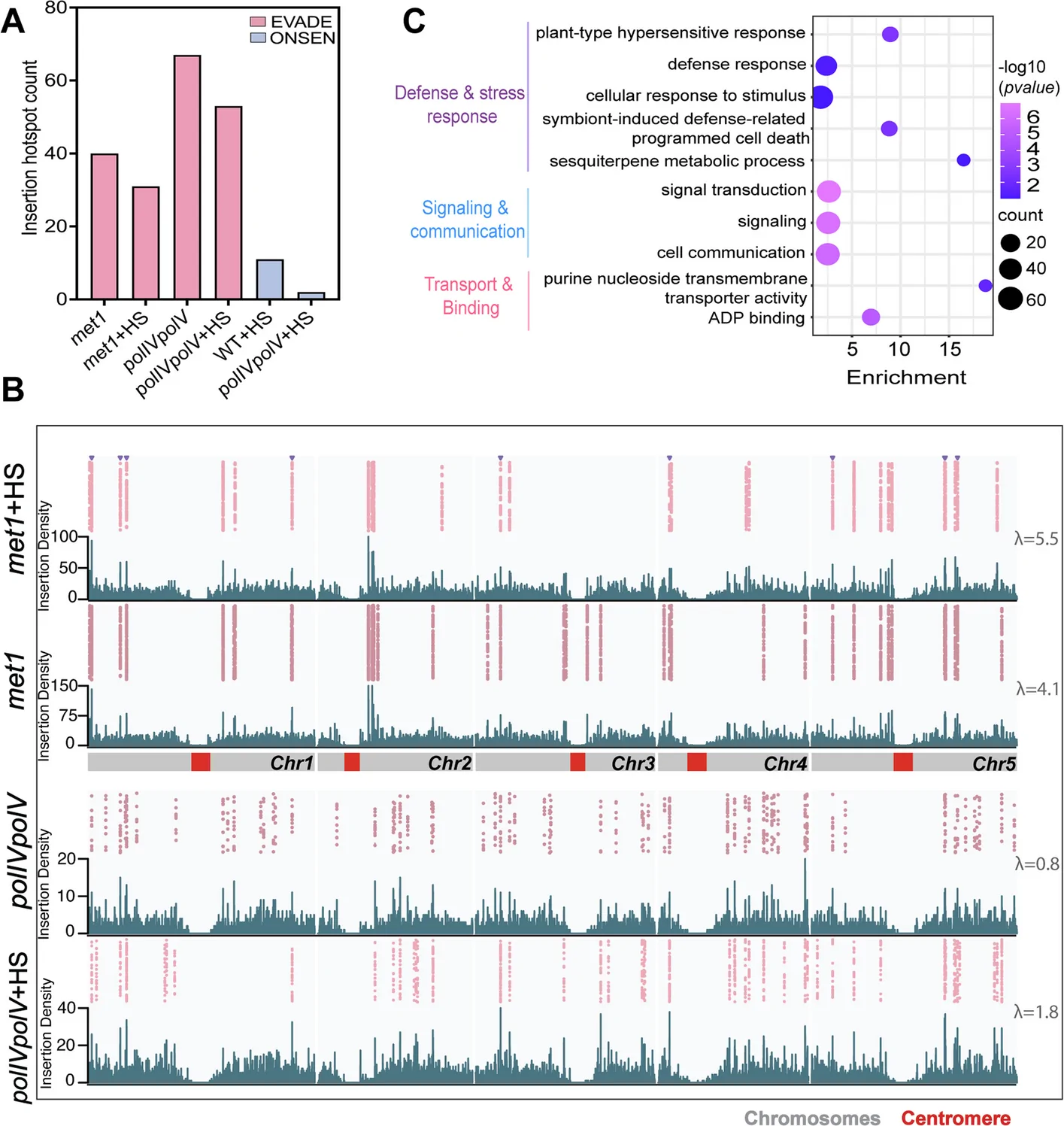
Genomic hotspots characterized by recurrent de novo insertions** A** Somatic insertion hotspots identified for EVADE and ONSEN. **B** Distribution of insertion hotspots of EVADE across the *Arabidopsis* genome (upper panel). Each red dot represents an insertion in a 10 kb hotspot cluster. Insertion density plotted for 10 kb genomic windows is shown in the lower panel. The expected number of insertions per 10 kb bins (λ) under null hypothesis are indicated for each sample. Hotspots shared between all samples are indicated by purple triangles. **C** Gene ontology analysis of genes co-localized in the EVADE hotspots. Enriched GO terms under biological function and molecular processes have been shown

We next investigated whether specific genomic “hotspot” regions are preferentially selected for recurrent somatic insertions. For each TE family and experimental condition, “insertion hotspots” were characterized as genomic regions (10 kb bins) with insertion frequencies 10 times higher than expected by chance and/or exhibiting statistically significant enrichment under Poisson test. For EVADE, we detected 40 local hotspots in *met1*, 31 in *met1* + HS, 67 in *polIVpolV* and 53 in *polIVpolV* + HS, all of which showed both the ≥ 10-fold higher insertion counts and significant enrichment based on Poisson test (FDR < 0.05; Fig. 5A; Additional Data 5). The average frequencies of the EVADE somatic integrations in the hotspot clusters ranged from 55 to 139 in *met1*, 41 to 92 in *met1* + HS, 8 to 20 in *polIVpolV* and 18 to 37 in *polIVpolV* + HS (Fig. 5B). Notably, 10 genomic hotspots were persistently targeted for EVADE somatic insertions across all samples and mutant lines, suggesting stable preferential targeting of these genomic sites (Fig. 5B). In addition, 25 and 26 EVADE hotspots were shared between the control and heat-stressed samples of *met1* and *polIVpolV*, respectively. For ONSEN, we identified 11 hotspots in heat-stressed samples of wild-type plants based on the 10-fold enrichment criterion, with the corresponding bins containing 4–5 insertions. In heat-stressed *polIVpolV plants,* two hotspots were detected based on significant Poisson enrichment (FDR < 0.05), each containing 9 insertions (Fig. 5A; Additional file 5). No ONSEN hotspot was shared across different samples (Additional file 2: Fig. S18). Notably, no hotspot loci were shared between EVADE and ONSEN. Overall, the hotspots from both TE families were uniformly distributed across all five chromosomes (Fig. 5B; Additional file 2: Fig. S18). No hotspot clusters were identified for AtCOPIA21.

To gain insights into the biological relevance of these somatic TE hotspots, we performed gene ontology (GO) analysis on the genes co-localized within the EVADE hotspots. The principal GO terms enriched under biological processes were associated with pathways involved in defence & stress response, as well as signal & communication while for the molecular function, ADP binding and transporter activity emerged as the predominant classes (Fig. 5C). These results mirror earlier studies showing a preferential association of EVADE germline insertions in environmentally responsive genes [41, 56]. No GO categories were enriched for genes within ONSEN hotspot sites.

### Assessing predisposition of environmentally-responsive regions as biased de novo integration sites

The specific enrichment of environmentally responsive genes within the insertion hotspots prompted us to investigate if such regions are predisposed for enhanced de novo TE integrations. We explored our hypothesis by analysing two functional regions in the *Arabidopsis* genome which are actively modulated by environmental cues: Resistance (R) genes, which mediate plant defence against pathogens [57] and biosynthetic gene clusters that contain multiple genes with roles in specialized metabolic pathways and show co-regulation upon environmental and stress cues [58]. The insertion density of de novo somatic transposition overlapping with these regions was analysed and compared to a similar set of randomly generated loci, sampled 1000 times by applying permutation tests.

**Fig. 6:**
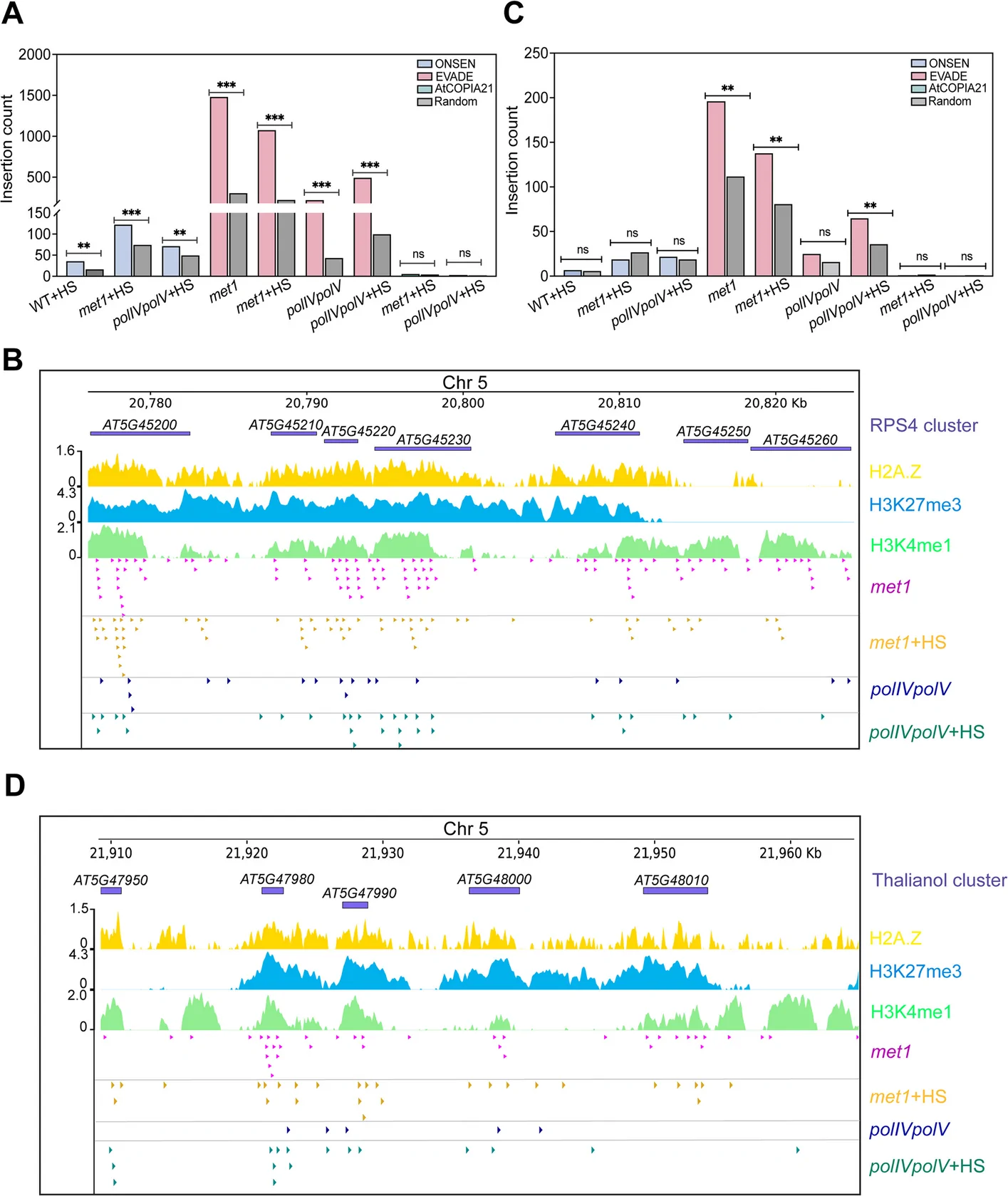
Environmentally-responsive regions as biased de novo integration sites. **A**, **C** De novo somatic insertions of the three TE families overlapping with R genes (**A**) and biosynthetic genes (**C**). Permutation test was applied to evaluate level of significance (***p < 0.001; **p < 0.01; ns-non-significant). **B**, **D** Genome browser profiles showing distribution of de novo somatic insertions of EVADE in representative R gene cluster, RPS4 (**B**) and thalianol biosynthetic gene cluster (**D**) of *A. thaliana*. Tracks showing the distribution of genes (purple) and epigenomic marks including H2A.Z (yellow), H3K27me3 (blue) and H3K4me1 (green) are presented. Each arrowhead in the four tracks below indicates a unique somatic insertion event of EVADE identified in samples from mutant lines. Genomic coordinates have been indicated for each plot

Focusing on 170 R genes in *A. thaliana* [57, 59], we detected high rates for somatic insertions of both EVADE and ONSEN (*p* < 0.01, Permutation test; Fig. 6A; Additional file 6). The SSI4 and RPS4 gene clusters exhibited the highest frequencies of EVADE somatic insertions, with a total of 370 and 199 independent integrations across all samples (Fig. 6B; Additional file 6). Reflecting patterns observed for R genes, somatic insertions for EVADE—but not ONSEN—were substantially over-represented in the 62 biosynthetic genes in *met1* and heat-stressed *polIVpolV* (*p* < 0.01, Permutation test; Fig. 6C, D; Additional file 7). In fact, the epigenomic profile of both the RPS4 and thalianol clusters showed strong enrichment for the same marks (H3K27me3, H2A.Z, and H3K4me1) that were associated with the general EVADE insertion bias (Fig. 6B, D).

Clusters of both R genes and biosynthetic genes have previously been reported to be enriched for TEs [60–62]. To test if other regions of high TE load are preferentially targeted by de novo TE insertions, we examined and contrasted the distribution of somatic insertions within the KNOT region—a 3D nuclear structure that has been demarcated as a preferred integration hotspot for invading TEs [63, 64]. However, we found no enrichment for new somatic insertions of all three TE families in the KNOT regions (Additional file 2: Fig. S19; Permutation test, *p* > 0.05). Our findings show that resistance and biosynthetic genes function as preferential integration sites for TEs, therefore suggesting that TEs may have a critical role in shaping their evolution and plastic functionality.

## Discussion

In multicellular organisms, somatic cells do not share identical genomes, instead they exhibit genomic mosaicism with significant differences between cells [65]. The genome-wide contribution and impact of TEs in this mosaicism has been mostly studied in animals, focusing on brain cells and cancerous or ageing tissues [10, 11, 13, 16, 18]. Here, our study provides direct evidence for TE-mediated genomic variation in somatic cells of a plant at an unprecedented scale. By identifying hundreds of thousands of de novo somatic transposition events (Fig. 2), we show that the transcriptional activity of TEs rapidly translates to integration of TE copies into the genomes of somatic cells, thus generating genetic diversity between cells. Although transient, the variability contributed by somatic TE insertions could influence intra-organismal cell identity and tissue functionality, in addition to imparting detrimental or useful effects. For example, evidence that somatic TE insertions could confer beneficial phenotypic variation in plants is reflected by the vast diversity observed in the bud sport somatic chimeras of clonally propagated crop species such as apple and oranges [27, 28, 66, 67]. Given that our sampling consisted only of a few leaves from young seedlings, we speculate that somatic transposition in the plant body is an explosive and potentially recurring event when conditions are favourable in both natural populations and experimental conditions.

Recent work illustrates how stress-activated ONSEN elements can modulate the adaptive response of genes near a novel fixed insertion site [68, 69]. As we provide evidence that ONSEN and other TEs actively transpose to new genomic sites in somatic cells of Arabidopsis including wild-type plants, it is likely that de novo TE insertions also imitate this modulated stress response of proximal genes in somatic cells and thereby, transiently alter stress physiology in a spatio-temporally dynamic manner. The downstream effects of this modulation can be important for both crops and perennial species like trees, and also in species with high loads of recently active TEs that can collectively contribute en masse to somatic mosaicism. Very recently, one study in maize attempted to quantify levels of somatic transposition of the Mutator DNA transposon using ultra-deep short-read sequencing but with no analysis of their genome-wide genetic and epigenetic characteristics [70], while two studies in *A. thaliana* retrieved a small number of somatic insertions using different long-read sequencing approaches [71, 72]. Along with our findings, these studies underscore an emerging focus on investigating the dynamics of TEs in the soma of plants, warranting a systematic investigation across diverse plant systems.

Our work demonstrates that global somatic transposition rates as well as integration sites are TE-specific and influenced by the genetic background of the host. In line with previous reports highlighting EVADE as the most successful TE in generating a high frequency of germline insertions in Arabidopsis [41, 73], we observed extreme mobility of EVADE in somatic cells of epigenetic mutant lines (Fig. 2). For ONSEN, abundant new somatic insertions were recorded in heat-stressed samples of both wild-type and mutant Arabidopsis lines. Compared to EVADE and ONSEN, the somatic transposition rate of AtCOPIA21 was low in the studied mutants, which is consistent with TE families responding with varying levels to induction triggers [74]. For all families, loss of CG methylation in *met1* imparts greater release of repression than in the double *polIVpolV* mutant*.* This disparity likely stems from the central role of *met1* in maintaining TE suppression, compared to the locus-specific and reinforcing action of *polIVpolV* and RdDM [75]. Furthermore, loss of *met1* not only attenuates DNA methylation over CG sites but also modifies several chromatin signatures necessary for maintenance of genome-wide TE repression, which could explain the pronounced activation of TEs observed in this mutant [76].

Delimiting the factors that guide TEs to their initial integration sites is fundamental to TE biology but also crucial for our broader understanding of genome dynamics. In the absence of selection during somatic transposition, we found that all three TE families showed a general de novo integration preference for the euchromatin-rich chromosomal arms and depletion from constitutive heterochromatic regions in the peri/centromere (Fig. 3)—a pattern that aligns with previous observations for non-reference segregating insertions for these TE families [41, 44, 68]. At the regional scale, we observed deviating integration site preferences with EVADE showing much higher integration preference for genes compared to ONSEN and AtCOPIA21. At the epigenetic level, previous reports have demonstrated the critical role of H2A.Z and H3K27me3 chromatin marks in shaping the preferred integration niche for germline insertions of Copia families [41, 68]. Here, we also observed that de novo somatic insertions of EVADE and ONSEN were enriched over H2A.Z and H3K27me3 marked regions, albeit with notably stronger enrichment for EVADE (Fig. 4). In addition, both families have a strong integration preference for H3K4me1, which is a conserved chromatin mark enriched over active genes and associated with transcription [77]. H3K4me1 is also actively involved in the recruitment of the DNA repair machinery and has been correlated with lower mutation rates in Arabidopsis [78, 79], which raises the possibility that H3K4me1-labelled sites are inherently susceptible to new TE integrations and, therefore, are targeted by the DNA repair machinery to facilitate removal of new integrations and protect genome integrity.

In contrast to ONSEN, EVADE somatic insertions were depleted from active chromatin marks other than H3K4me1 and from areas of open chromatin. This finding is consistent with TEs occupying different target site niches at the local epigenetic level, helping to avoid conflict for space in the genome ecosystem. This spacing may increase mutational load, depending on epigenetic state and environmental condition. Indeed, the EVADE and ONSEN hotspots showed no overlap, yet some genome areas were highly susceptible to insertions of both families (Fig. 6). For example, within R gene clusters, we observed hundreds of recurrent and independent insertions of EVADE and ONSEN TEs. This may suggest that R genes are in fact acting as sponges for new somatic TE insertions, which may drive the diversification of these disease resistance loci, and may modulate their regulatory properties. Intriguingly, EVADE expression is elevated upon pathogen stimulation [80, 81], and so may directly affect R gene cluster architecture and expression in individual cells and tissues [81, 82]. Our findings demonstrate that R gene clusters are predisposed for TE integrations and that this feature may be a key element in the adaptability of defence genes to changing pathogen threats.

## Conclusions

In summary, by documenting the accumulation en masse of TE insertions in the soma of *Arabidopsis*, we provide evidence for the plasticity of plant genomes at the cellular level but also for the unique integration site preferences of TEs independent of selection. We speculate that these features enable host genomes to generate genetic diversity at genomic sites of need, which leads to genetic cellular plasticity at times of TE mobilisation. Our work establishes that studying somatic transposition is a reliable and powerful approach towards understanding TE dynamics, and, thereby, genome function and evolution.

## Methods

### Plant materials and experimental growth conditions

*A. thaliana* wild type Col-0 and mutant plants were grown on ½ Murashige and Skoog (MS) medium in a growth chamber at 22 °C under long day conditions (16 h light, 8 h dark) following an initial stratification for 2 days at 4 °C. Details of the mutant alleles used in the study are provided in the Additional file 1: Table S1. All the wild type and mutant alleles were in Col-0 ecotype background. Young leaves harvested from a pool of ~ 100 individual seedlings were used for all the experiments.

### Heat stress regimes for AtCOPIA78 (ONSEN) induction

For heat stress application, the 10 days old seedlings grown on MS medium were subjected to three variable heat regimes to quantify ONSEN induction (Fig. 1A, Additional file 2: Fig. S1). A high temperature of 37 °C was used for all heat-stress treatments.

(i) For the present study, we define long-duration heat stress as high temperature exposure that spanned over 8 continuous days. The WT seedlings pre-treated to low temperature (6 °C) were allowed to grow for 8 days at high temperature. At every 24 h, leaf samples were harvested to monitor ONSEN activity. Additionally, at each indicated time point, a similar set of HS seedlings were transferred to normal growth conditions (22 °C, 16 h light, 8 h dark cycle) for a 48 h recovery period (Fig. 1A).

(ii) Effect of low temperature before heat application on ONSEN activation was tested by exposing the WT seedlings to high temperature of 37 °C for 48 h with or without prior exposure to chilling temperature of 6 °C for 24 h (Additional file 2: Fig. S1).

(iii) In the third variation of the applied heat regime, cold-treated WT and mutant seedings were exposed to high temperatures for up to 48 h. Samples were collected and analysed at intervals of 0, 6, 12, 24 and 48 h during the heat stress period (Fig. 1A).

Leaf tissues were harvested immediately after completion of specified timepoints for each treatment and frozen in liquid nitrogen. The samples were stored at −80 °C until further use.

### RNA extraction and qRT-PCR for TEs transcription abundance

Total RNA was extracted from harvested tissues using Trizol reagent (Sigma) and DNase-treated with Turbo DNA-free kit (Thermo) following manufacturer’s instruction. Starting with 1 µg of total RNA, complementary DNA (cDNA) was synthesized with iScript cDNA synthesis kit (Biorad) according to manufacturer’s instructions. TE transcript abundance was evaluated through qRT-PCR using PowerUp SYBR Green Master Mix (Applied Biosystems). TE primer sequences are listed in Additional file 1: Table S5. Expression levels were determined relative to the housekeeping control gene (18S rna or ACT2), applying the 2–∆∆*Ct* method. Mean and standard deviations were inferred from three biological replicates for all experiments unless stated otherwise.

### DNA extraction

For eccDNA assays and whole genome sequencing, total DNA was extracted from sampled tissues using standard CTAB method [83]. For TEd-seq, high molecular weight DNA was prepared after nuclei isolation from harvested tissue. Briefly, 0.5gm of leaf tissue was homogenized in a mortar and pestle using liquid nitrogen. ~ 20 ml of chilled nuclei isolation buffer (NIB: 20 mM Tris–HCl pH 7.8, 250 mM sucrose, 5 mM MgCl2, 5 mM KCl, 40% Glycerol, 0.25% Triton X-100, and 0.1% β-mercaptoethanol) was added to the grounded powder. The thawed homogenate was filtered through four layers of Mira cloth and a 40-μm-thick nylon mesh and the filtrate was centrifuged at 6000xg for 15 min at 4 °C. The pellet was gently washed with 20 ml of NIB and the samples were incubated on ice for 10 min followed by centrifugation at 6000xg for 15 min at 4 °C. This step was repeated 4 times or until a clear pellet was obtained. Next, the nuclei pellet was resuspended in prewarmed Carlson lysis buffer (100 mM Tris pH 8, 2% CTAB, 1.4 M NaCl, 1% PEG, 20 mM EDTA) along with 25 μl of β-mercaptoethanol and 40 μl of RNaseA. The samples were incubated at 65 °C for 1 h with intermittent mixing. Post incubation, the contents were allowed to reach room temperature, an equal volume of chloroform:isoamyl alcohol mix (24:1) was thoroughly mixed with it and centrifuged at 6000xg for 10 min at 4 °C. The upper aqueous layer was extracted in a fresh tube and another round of chloroform:isoamyl alcohol treatment was performed. DNA was precipitated with one-tenth volume of 3 M sodium acetate (pH 5.2) and chilled isopropanol. The DNA pellet was washed with 70% ethanol, air dried, and dissolved in molecular biology grade water. DNA quality was assessed through electrophoresis on 1% agarose gel and Nanodrop 2000 (Thermo Scientific) measurements. DNA amount was quantified by dsDNA High Sensitivity Assay kit and Qubit Fluorometer (Invitrogen).

### Detection of extra-chromosomal circular DNA (eccDNA) and inverse PCR

To selectively remove linear DNA and enrich eccDNA, 1 µg of RNased DNA was digested with 1 µl of Plasmid-Safe ATP-dependent DNase (Lucigen) in a total reaction volume of 50 µl and incubated at 37 °C for 16 h. Complete removal of linear DNA was ensured by adding an additional 1 µl of DNase and ATP to the reaction mixture after 16 h. This step was repeated twice at 2 h interval resulting in a total digestion duration of 20 h after which the DNase activity was terminated by incubation at 70 °C for 30 min. eccDNA was retrieved by overnight precipitation with 0.1 volume of 3 M sodium acetate (pH 5.2), 2.5 volumes of pure ethanol and 1 µl of glycogen (Thermo) at −20 °C. The precipitate was centrifuged at 14,462xg for 1 h at 4 °C followed by pellet washing with 70% ethanol. The air dried eccDNA pellet was resuspended in TempliPhi Sample buffer and rolling circle amplification was performed using TempliPhi Amplification kit (Cytiva) by incubation at 28 °C for 65 h. The reaction was terminated by inactivating enzyme at 70 °C for 30 min. The enriched eccDNA product was used as template for inverse PCR to selectively amplify TE-specific products using outward primers (Additional file 1: Table S5). The eccDNA circle (Chr2: 3,416,272-3417604_ +; hereafter designated as eccUni) identified ubiquitously in multiple *Arabidopsis* tissues was used as a positive control for eccDNA profiling [46]. Inverse PCR products of AtCOPIA78 (ONSEN) and AtCOPIA93 (EVADE) were cloned into pGEMT vector (Promega) and sequence was verified through Sanger sequencing.

### Whole genome sequencing with Illumina and detection of de novo transposon insertion from resequencing data

HMW genomic DNA was used for preparation of Illumina short read sequencing libraries with an insert size of 350 bp using TruSeq DNA Library Prep kit. The libraries were sequenced on NovaSeq6000 platform in a paired end (PE) mode (2 X 150 bp) at Novogene, UK to achieve an expected genome coverage depth of over 100 folds. The Col-CEN, telomere-to-telomere assembly of *Arabidopsis* Col-0 accession and its associated annotations [53] were used consistently throughout the study for all genomic-based analysis. The quality assessment of the raw reads was performed with FASTQC (https://github.com/s-andrews/FastQC). Trimmomatic [84] was used to filter the low-quality and trim adapter-contaminated reads applying the following parameters ILLUMINACLIP:TruSeq3:2:30:10 LEADING:20 TRAILING:20 SLIDINGWINDOW:4:20 and MINLEN:36. The consensus TE sequence library for Col-CEN was derived by lifting over the TE annotation from Col-CC reference assembly (https://github.com/oushujun/TAIR12-TE) using liftoff version1.6.3 [85]. The retrieved TE library was used as an input for TEMP2 v 0.1.6 [48] to detect de novo somatic TE insertions through module insertion at default settings. TEMP2 output classify the obtained non-reference insertions into three main classes: *1p1 insertions* have read support at both ends of a TE, 2p insertions have 2 or more read support only at one end of TE while singleton insertions are supported by a single read. 1p1 and 2p insertions are regarded as fixed heritable insertions while singletons as de novo somatic events. We re-classified the obtained de novo somatic insertions based on the following criteria: The insertions (classified as “1p1” and “2p” in TEMP2 pipeline) where the coverage was less than 5 reads were classified as de novo somatic insertions along with those supported by singletons. Further details of WGS libraries are provided in Additional file 1: Table S6 [51].

### Transposable element display sequencing (TEd-seq)

TE-specific enrichment libraries were prepared by optimizing the TEd-seq protocol [35] for three LTR-TE families: ONSEN, EVADE and AtCOPIA21 using NEBNext® Ultra™ II DNA Library Prep Kit for Illumina® (NEB). Briefly, HMW genomic DNA was extracted from leaves of a pool of ~ 100 Arabidopsis seedlings. To obtain fragmented DNA in a size range of 300–700 bp, ~ 2 µg of genomic DNA dispensed in 100 µl TE buffer was sonicated for 8 cycles of 30 s treatment/30 s delay in Covaris M220 (Peak power 75 W, Duty factor 10 and 200 cycles per burst). End repair and A-tailing of 1 µg sheared DNA was performed using NEBNext Ultra II End Prep Enzyme Mix in a total volume of 60 µl (NEB). TEd-seq custom adapters (Additional file 1: Table S7) were ligated using NEBNext Ultra II ligation Master Mix and NEBNext Ultra II ligation enhancer followed by size selection with AMPure XP Beads (Beckman Coulter). First round of selective TE-fragment enrichment PCR (primary PCR) was performed with outer TE-specific primer (TE*_rev; * indicate TE family; Additional file 1: Table S7) and adapter-specific primer in a total volume of 50 µl with the following cycling conditions: 98 °C for 30 s followed by 20 cycles of 98 °C for 10 s, 61 °C for 75 s and a final cycle at 61 °C for 5 min. The PCR products were cleaned and size-selected using 0.9X ratio of AMPure XP Beads. The cleaned PCR product was used as a template for a second round of nested PCR using an inner TE primers mix with Illumina P5 overhang (P5_TE*; * indicate TE family) and P7 adapter primer with index in a total volume of 25 µl. The amplification conditions used are: 98 °C for 30 s, *n* cycles of 98 °C for 10 s, 61 °C for 75 s, *m* cycles of 98 °C for 10 s, 72 °C for 75 s and final cycle at 72 °C for 5 min (*n* and *m* represents specific PCR cycle number optimized for each TE family; Additional file 1: Table S7). The final DNA library was subjected to two-sided size selection to capture fragments in range of 200–600 bp with AMPure XP beads using sequential ratios of 0.67X and 1X, and eluted in a final volume of 30 µl TrisHCl (10 mM). Illumina short-read sequencing was performed at Novogene,UK on NovaSeq platform in a paired end (PE) mode (2 X 150 bp). For pooled multiplexed TEd-seq libraries, primers of different targeted TEs were mixed in equimolar concentrations. Library details are given in Additional file 1: Table S3.

### Computational analysis with deNOVOEnrich

The TEd-seq bioinformatic pipeline as described in the original reference [35] is intended to capture non-reference segregating insertions. To efficiently identify and profile rare somatic transposition events in addition to non-reference segregating TE insertions from TEd-seq data, we have designed a new computational pipeline, deNOVOEnrich [50]. The general principles and overall workflow of the deNOVOEnrich pipeline is presented in Additional file 2: Fig. S6.

For deriving transposition rate across different samples, we normalized the raw data [51] for each TE family by downsampling reads in the samples to the scale of the sample with least number of TE-specific reads. This normalization strategy via downsampling was adopted to ensure all samples are compared at an identical effective sequencing depth and detection sensitivity, avoiding the bias introduced by the heterogeneous TE-read coverage in the sample libraries. Additionally, it also allowed us to estimate minimum TE-specific read depths that are sufficient to robustly detect somatic insertions in our samples. The first step of deNOVOEnrich pipeline involves stringent identification of TE-specific paired-end reads from raw TEd-seq data. This is achieved by using as bait a ~ 50—60 bp terminal sequence that is extending from the TEd-seq primer binding site to the 5’ end of the targeted TE. This sequence is supplied to cutadapt [86] as the functional equivalent of an adapter. This allows us to sensitively detect reads that contain this TE-specific segment while filtering out non-specific amplification or chimeric fragments from the analysis. Subsequently, fastp [87] is applied to identify low quality reads, sequencing artefacts (including reads with polyG tails associated with NovaSeq technologies) and short read fragments (< 20 nt in length) producing a final set of high confidence TE-specific reads. These identified TE-specific reads are then retrieved from the original raw data with seqtk (https://github.com/lh3/seqtk) based on IDs and used for further processing after adapter trimming with trimmomatic with the parameters: ILLUMINACLIP:TruSeq3:2:30:10 LEADING:3 TRAILING:3 SLIDINGWINDOW:4:15 and MINLEN:20 [84]. To further increase alignment accuracy and detection of TE-genome split junction, we generated DNA contigs by concatenating read pairs into single reads (hereafter referred to as “long DNA contigs”) with a minimum overlap of 5 base pairs at their ends and keeping the contig lengths in a range between 50 bp (-n) to 300 bp (-m) using PEAR [88]. The long DNA contigs are then mapped onto the reference genome with bowtie2 [89] allowing split-read mapping in local mode (--local, –very-sensitive). Subsequently, alignments on the reference genome are filtered to retain only those mapped reads that align at a unique location in the genome and harboured at least 40 nucleotides of soft-clipped sequence while rejected reads that mapped on multiple locations and/or lacked sufficient split support. We used a score-based filter to identify multi-mapped reads. Specifically, we compared the primary alignment score (AS) of a read to its secondary score (XS). A read was considered ambiguously multi-mapping and removed from further analysis when the score difference between its primary and secondary alignment was less than 20%, corresponding to a secondary to primary alignment score ratio of ≥ 0.8. PCR duplicates were marked with picard MarkDuplicates (http://broadinstitute.github.io/picard/). The long DNA contigs were also mapped separately on the TE extremity sequence (that includes the region from the primer site to 5’ extremity end) in local mode with bowtie2. Mapping on the TE extremity sequence orthogonally checks that the clipped part of the read is genuinely contributed by the target TE, avoiding off targets from the genome. Next, we preserved split contigs by establishing shared read homology between the target TE and reference genomic sites using Readtagger with default parameters [10]. Using the SAM tags assigned by the Readtagger, TE-specific tagged reads that mapped to both the reference genome and targeted TE sequences were retained. These TE-tagged alignments which are indicative of TE:genome break sites were then used to determine *bona-fide* non-reference TE insertion sites in the genome.

TE insertions were mainly classified into two categories: de novo somatic insertions and non-reference segregating insertions, depending on the read depth at the putative TE integration sites. Frequency distribution of the read coverage for insertion sites was examined to choose the thresholds for defining somatic and non-reference segregating insertions in the datasets. For each TE family, we computed the read coverage distribution per insertion site which showed a consistent pattern: (i) a prominent peak dominated by a high number of cases with ≤ 5 reads coverage; and (ii) a steep decline in the number of insertions that has a coverage of > 5 reads; and (iii) in samples where non-reference segregating insertions are expected, a small increase in the number of insertions with a high coverage of ≥ 30 reads (Additional file 2: Figs. S7-9). Since somatic insertions occur in low frequency in single or few cells, which depends on the number of cell replication cycles during our experimental conditions, all insertions supported by a low total read depth of ≤ 5 including singleton reads were classified as de novo somatic insertions. TE insertions with a threshold coverage of ≥ 30 reads, supported by at least 5 unique contig reads (sharing the same start site but variable stop sites) and with at least 3 of the unique reads containing 3 or more PCR duplicates were classified as non-reference segregating insertions. Insertions with intermediate coverage between 5 to 30 reads were excluded from the analysis to enable confident assignment to somatic and non-reference segregating insertion categories.

Highly stringent filters were applied at each step of the pipeline to ensure removal of false positives and reduction of background noise signal. Firstly, TE insertions localized within 1 kb window of reference TE sites in the genome were discarded as previous studies have shown that these regions contribute to high rate of chimeric read generation leading to frequent misalignment [9]. Next, insertions within 50 bp window of mono-nucleotide genomic stretch or overlapping with organelle genomes were filtered to remove ambiguous alignment sites. Finally, for de novo somatic insertion events, any insertions identified at the same genomic coordinates across multiple samples were excluded from further analysis, as the likelihood of identical somatic insertion sites is highly improbable [90]. The source code of the pipeline is available at the GitHub repository [50]. Genomic distribution of de novo TE insertions was displayed through circular plot generated through Circos [91].

### Genomic and epigenomic features association for TE-targeted sites

Target site preferences of different TE families were studied by evaluating the overlap between TE insertions and several genomic feature datasets. To analyse integration preferences at the genomic regional level, the *Arabidopsis* genome was differentiated into following distinct regions: protein coding genes (PCGs), proximity to genes (500 basepairs upstream or downstream of PCGs), reference transposable elements and intergenic regions. The frequency proportion of de novo integrations overlapping in each genomic region was recorded. Further, insertions in the PCGs were classified into different genic categories in priority order of 5’UTR, 3’UTR, exons and introns. To determine the significance levels of insertion bias, permutation statistical test was applied for overlaps between somatic insertions and genomic compartments using randomizeRegions function in regioneR [92]. The degree of enrichment is indicated by permutation Z scores. Analysis of sequence motifs at the integration sites was performed by retrieving five nucleotides flanking both the upstream and downstream region of the insertion sites with bedtools utilities, *slop* and *getfatsa* [93]. The sequences were used as input for motif search using meme [94]. To determine local sequence patterns around the insertion sites, sequence logo was generated with WebLogo3 [95]. Epigenomic datasets from ChIP-seq and ATAC-seq (Additional file 1: Table S4) [96–103] were processed by first treating the raw reads for removal of low quality and adapter contaminated reads with Trim Galore with options –length 30 –quality 20 –stringency 1 -e 0.1 (https://github.com/FelixKrueger/TrimGalore) [104]. The high-quality reads were aligned to Col-CEN reference genome with bowtie2 using parameters: –end-to-end –sensitive. PCR duplicates were removed by samtools markdup. For ChIP-seq, peak calling was performed using macs3 with the following options -q 0.00001, -f BAMPE [105]. Depending on the epigenetic mark, either broad or narrow peak calls were used for evaluating its association with de novo insertions based on overlapping frequency. For ATACseq, findPeaks function of HOMER was used for calling peaks applying following parameters: -region -size 200 -minDist 202 -gsize 135,000,000 -tbp 0 (http://homer.ucsd.edu/homer/). Normalization and visualization of ATAC profiles was performed using deepTools [106]. Metagene plots of epigenomic features were generated using *computeMatrix*, *plotProfile* and *plotHeatmap* utilities of deepTools. For DNA methylation, raw reads from whole genome bisulphite data (BS-seq) [107] were processed to trim off adapters and remove low-quality reads using TrimGalore v 06.10 [104]. The filtered reads were then aligned to the reference Col-CEN assembly using Bismark with default parameters followed by deduplication [108]. Context-wise differentially methylated regions (DMRs) were called with methylKit [109] based on sliding window analysis with a window size of 200 bp and a step size of 50 bp. The genomic windows exhibiting a methylation level difference of greater than 25 with at least 5 cytosines covered by minimum of 5 reads, and a threshold FDR (q-value) of < 0.01 were considered as DMRs. Context-wise hypo-methylated DMRs (hypo-DMRs) within 100 bp of each other were merged to generate the final set of DMRs. We next inferred gene-body methylation (GbM) following Muyle et al. [110]. Briefly, context-wise (CG, CHG and CHH) per-site cytosine methylation was deduced from Bismark genome-wide cytosine report. A one-tailed binomial test was applied to evaluate each cytosine using the non-conversion rate as error rate and corrected for multiple testing using Benjamini–Hochberg false discovery rate. Each cytosine site with adjusted *p-value* < 0.001 and with a minimum of 3 reads coverage was designated methylated. For each gene, methylation levels were quantified within the coding regions of the longest transcript and calculated separately for each context. The context-wise baseline methylation levels were used to perform a binomial test to evaluate the enrichment of CG, CHG and CHH methylation per gene followed by correction for multiple testing with Benjamini–Hochberg false discovery rate. A gene was classified as GbM if it harbours ≥ 20 CG sites, CG methylation was significantly higher than the baseline methylation levels (adjusted *p-value* < 0.05) and CHG and CHH methylation were depleted compared to the background (adjusted *p-value* > 0.05). We identified a total of 5,135 GbM genes in WT Arabidopsis.

### Insertion hotspots

TE hotspots with high insertion density were detected by dividing the *Arabidopsis* reference genome into equal-sized non-overlapping bins of 10 kb with bedtools makewindows followed by evaluation of insertion coverage per bin with bedtools coverage. Based on the hypothesis of no integration bias, the expected number of insertions per 10 kb bin (λ) was estimated as the mean number of insertions across all bins for each sample. We first identified genomic bins exhibiting at least a tenfold higher number of insertions than that is expected (λ) under a random distribution. Using sample-specific λ, we next evaluated the probability of observing the detected number of insertions or more in each bin through the Poisson cumulative probability test. The obtained *p*-values were adjusted for multiple testing using the Benjamini–Hochberg false discovery rate (FDR < 0.05). Genomic bins were designated as insertion hotspot if they fulfilled either the tenfold enrichment criterion, showed statistical significance in the Poisson test or both. Gene ontology enrichment analysis was performed for genes localized in insertion hotspots using PANTHER Classification System [111].

### Analysis of TE-populated genomic regions: R genes, biosynthetic genes and KNOT region

The Arabidopsis functional genomic regions including R genes [57, 59], biosynthetic genes [112] and KNOT-regions [63] were assessed for their pre-disposition efficiencies as potential sites for attracting new TE integrations. To assess TE integrations bias in these regions, loci were randomized while preserving their intrinsic structure and characteristics, and association analysis was performed based on permutation tests with regioneR [92]. For R genes and biosynthetic genes, all protein coding genes were provided as universe for resampleRegions functions. Genomic insertion distribution plots were generated with pyGenomeTracks [113].

## Supplementary Information

Additional file 1. Supplementary Tables S1-S7.

Additional file 2. Supplementary information 1 and Supplementary Figures S1-S20.

Additional file 3. Uncropped gel images from main and Supplementary Figures.

Additional file 4. Data 4. Somatic insertions from different TE families recorded through whole genome sequencing.

Additional file 5. Data 5. Somatic insertion hotspots identified for EVADE and ONSEN.

Additional file 6. Data 6. List of 170 R genes and number of somatic insertions observed in each gene.

Additional file 7. Data 7. List of 62 biosynthetic genes and number of somatic insertions observed in each gene.

## Data Availability

All sequencing data generated in the present work including whole genome sequencing and transposon display sequencing (TEd-seq) have been deposited in the NCBI Sequence Read Archive (SRA) (https://www.ncbi.nlm.nih.gov/) under the BioProject number PRJNA1270934 [51]. The source code for deNOVOEnrich and additional files employed for the analysis are available on Github: https://github.com/hAmbreen02/deNOVOEnrich [50] and archived on Zenodo under https://doi.org/10.5281/zenodo.20143080 [114], both under the MIT license. Details of the public databases used for the epigenomic analysis are provided in Additional file 1: Table S4.
