# Supplementary material for: Somatic mobility of transposons is explosive and shaped by distinct integration biases in Arabidopsis thaliana
Source: Genome Biol. 2026 Jul 4;27:298. doi: 10.1186/s13059-026-04149-0 (PMC13621724; doi:10.1186/s13059-026-04149-0)
Supplement: Supplementary file 1 [file 13059_2026_4149_MOESM3_ESM.pdf]

Somatic mobility of transposons is explosive and shaped by distinct integration biases in *Arabidopsis thaliana*

Heena Ambreen, Basile Leduque, Leandro Quadrana, R. Keith Slotkin, Alexandros Bousios, Hans-Wilhelm Nützmann

Uncropped agarose gel images presented in Figures – Figure 1E, Figure 1F, Fig. S3B, Fig. S5 and Fig. S20

Figure 1E

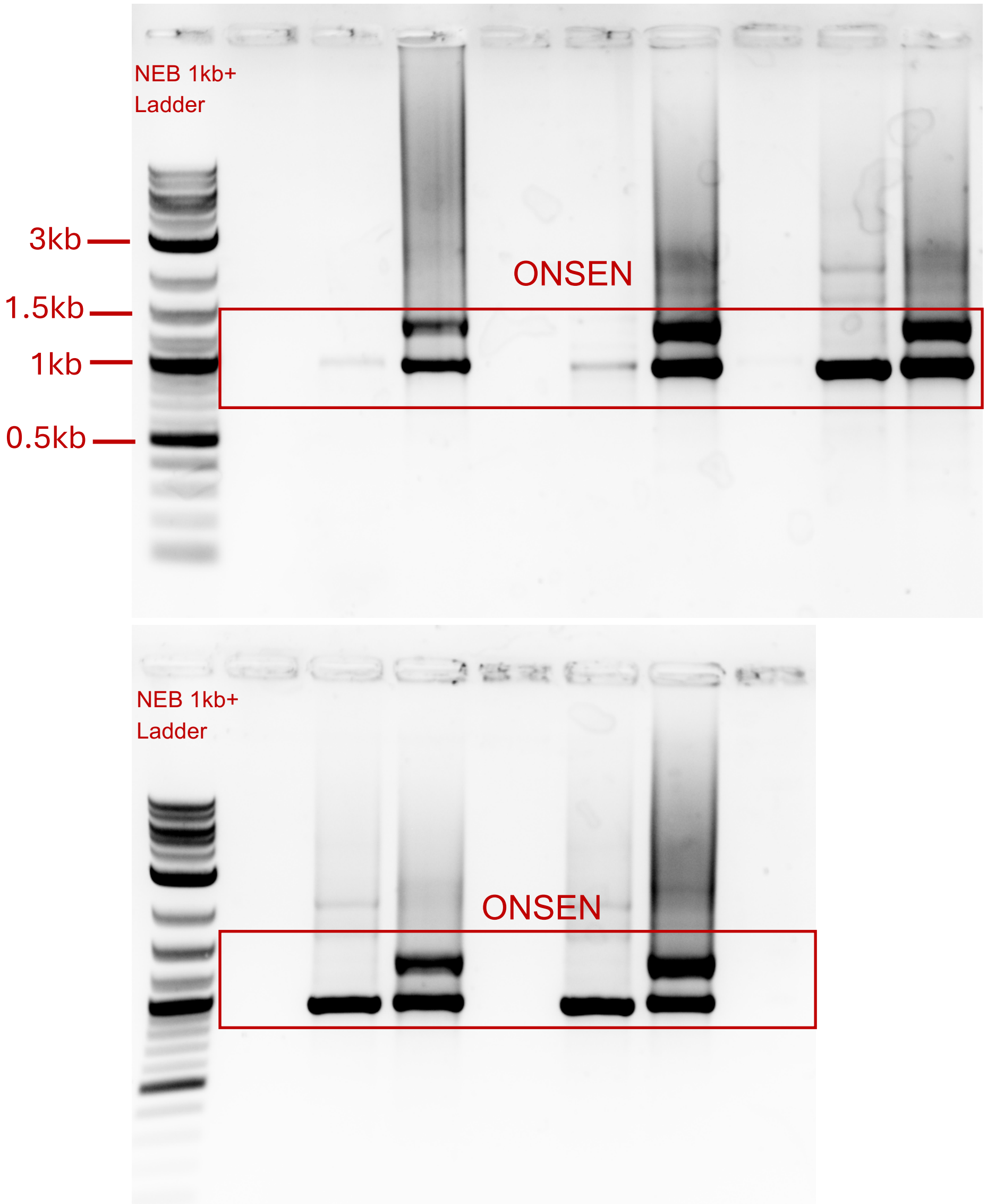

Figure 1E

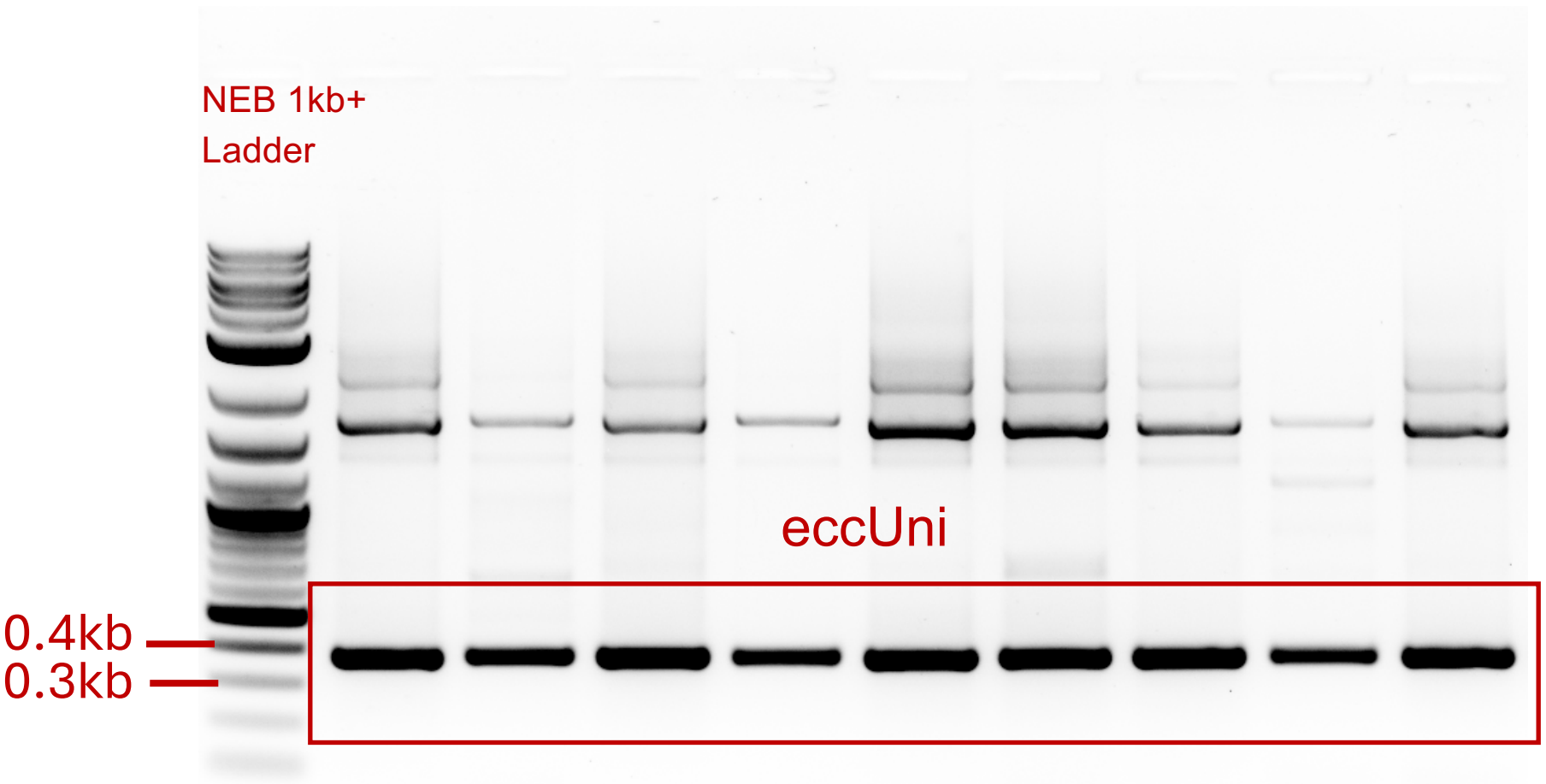

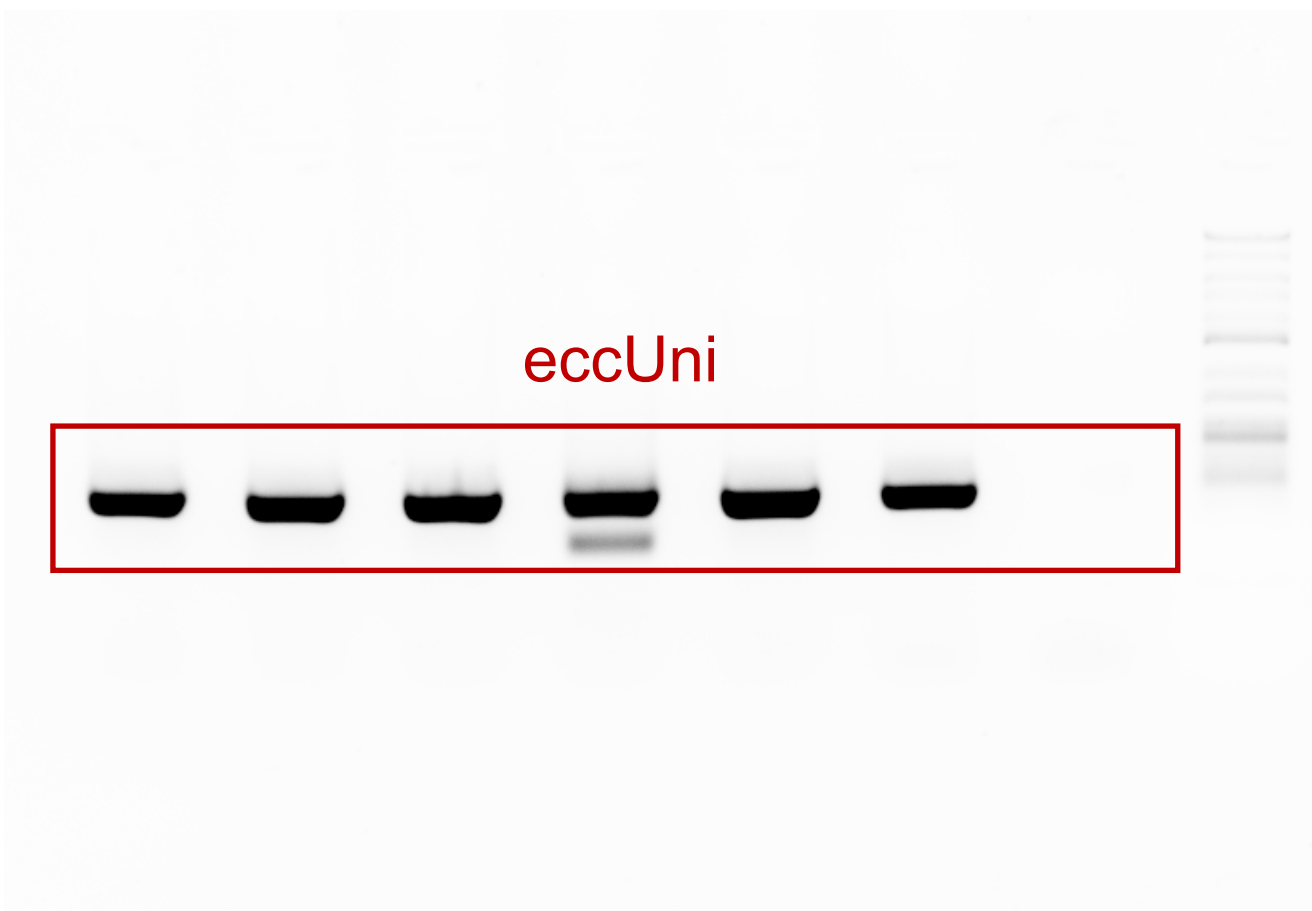

Figure 1F

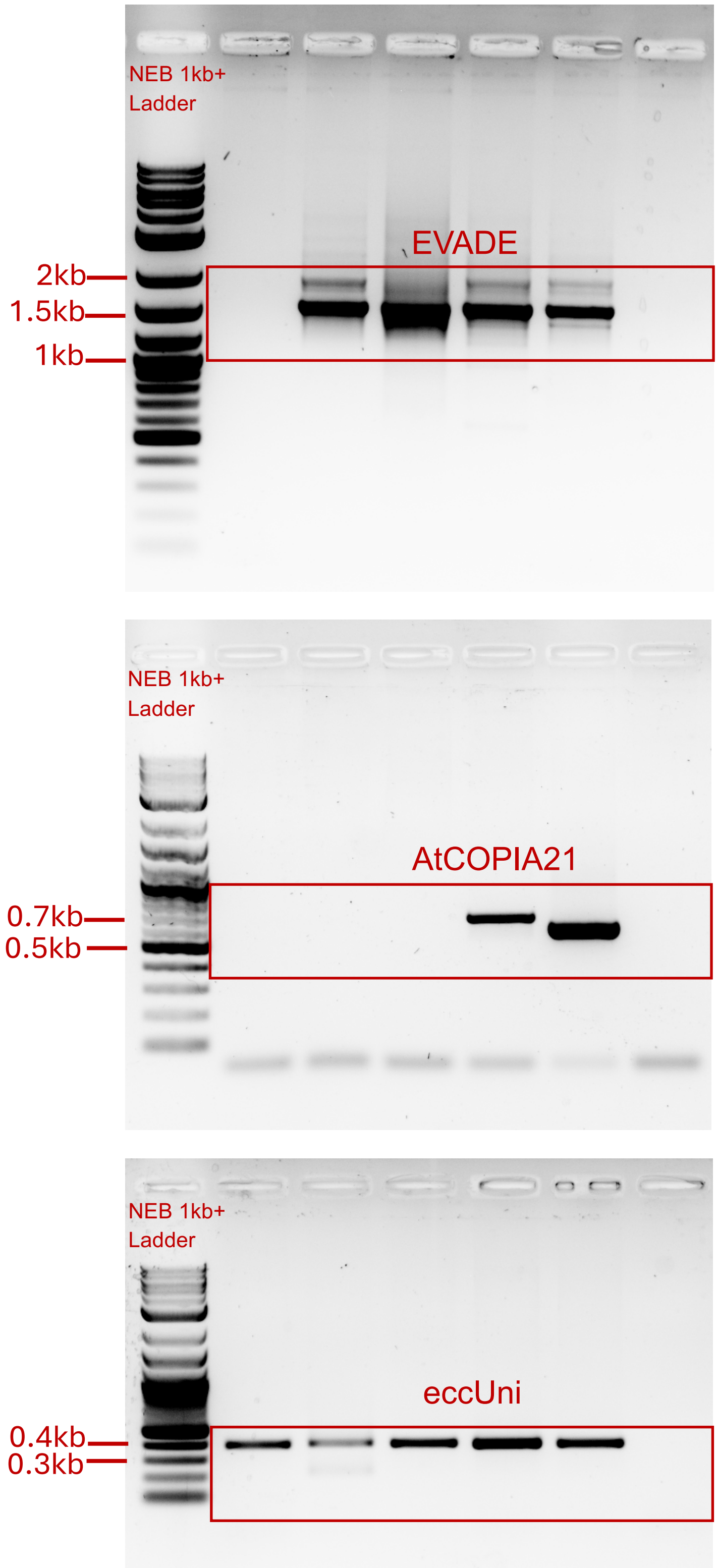
NEB 1kb+
Ladder
EVADE
2kb
1.5kb
1kb
NEB 1kb+
Ladder
AtCOPIA21
0.7kb
0.5kb
NEB 1kb+
Ladder
eccUni
0.4kb
0.3kb

Figure S3B

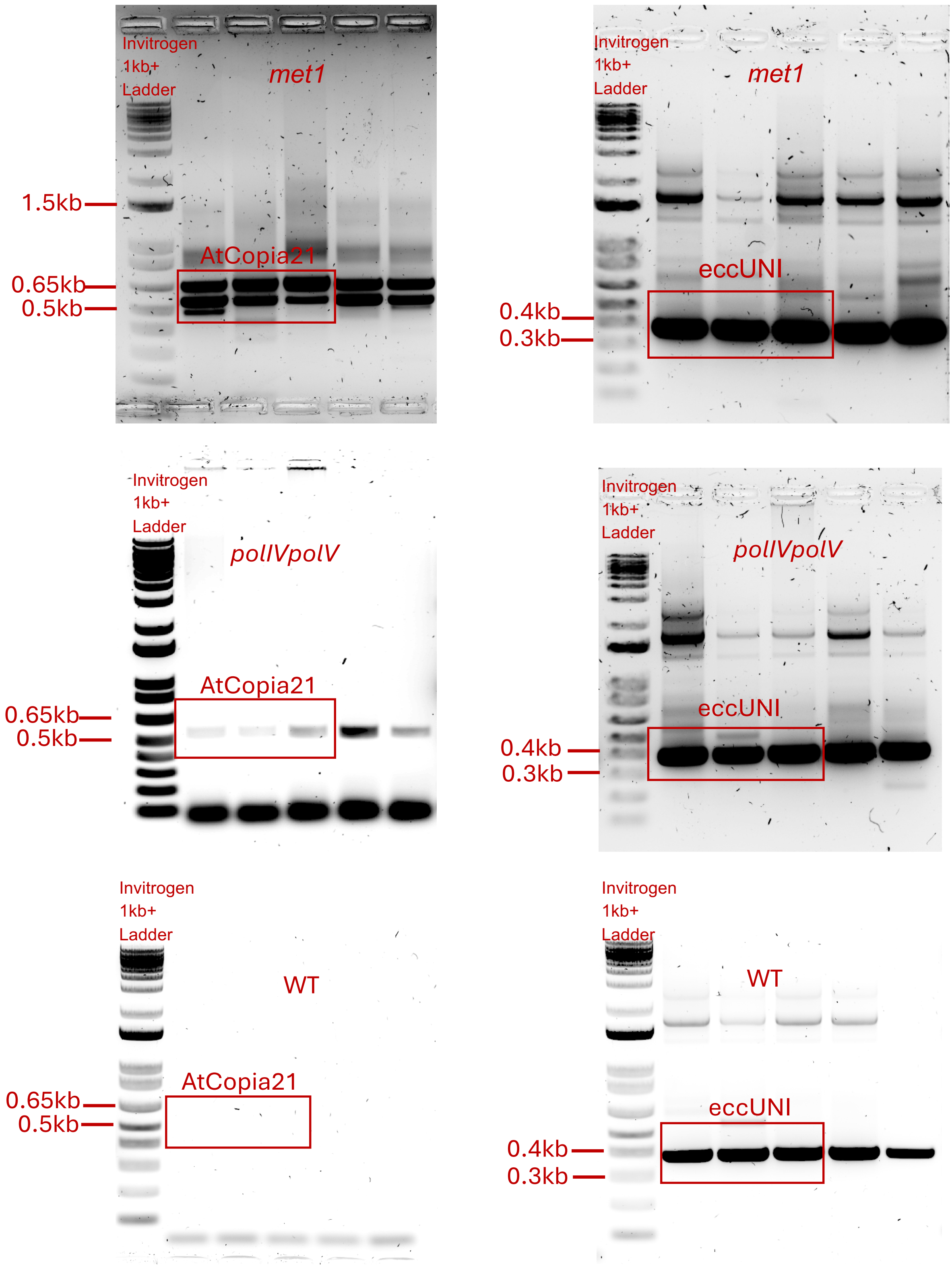
Invitrogen
1kb+
Ladder
met1
1.5kb
AtCopia21
0.65kb
0.5kb
Invitrogen
1kb+
Ladder
met1
eccUNI
0.4kb
0.3kb
Invitrogen
1kb+
Ladder
polIVpolV
AtCopia21
0.65kb
0.5kb
Invitrogen
1kb+
Ladder
polIVpolV
eccUNI
0.4kb
0.3kb
Invitrogen
1kb+
Ladder
WT
AtCopia21
0.65kb
0.5kb
Invitrogen
1kb+
Ladder
WT
eccUNI
0.4kb
0.3kb

Fig. S5

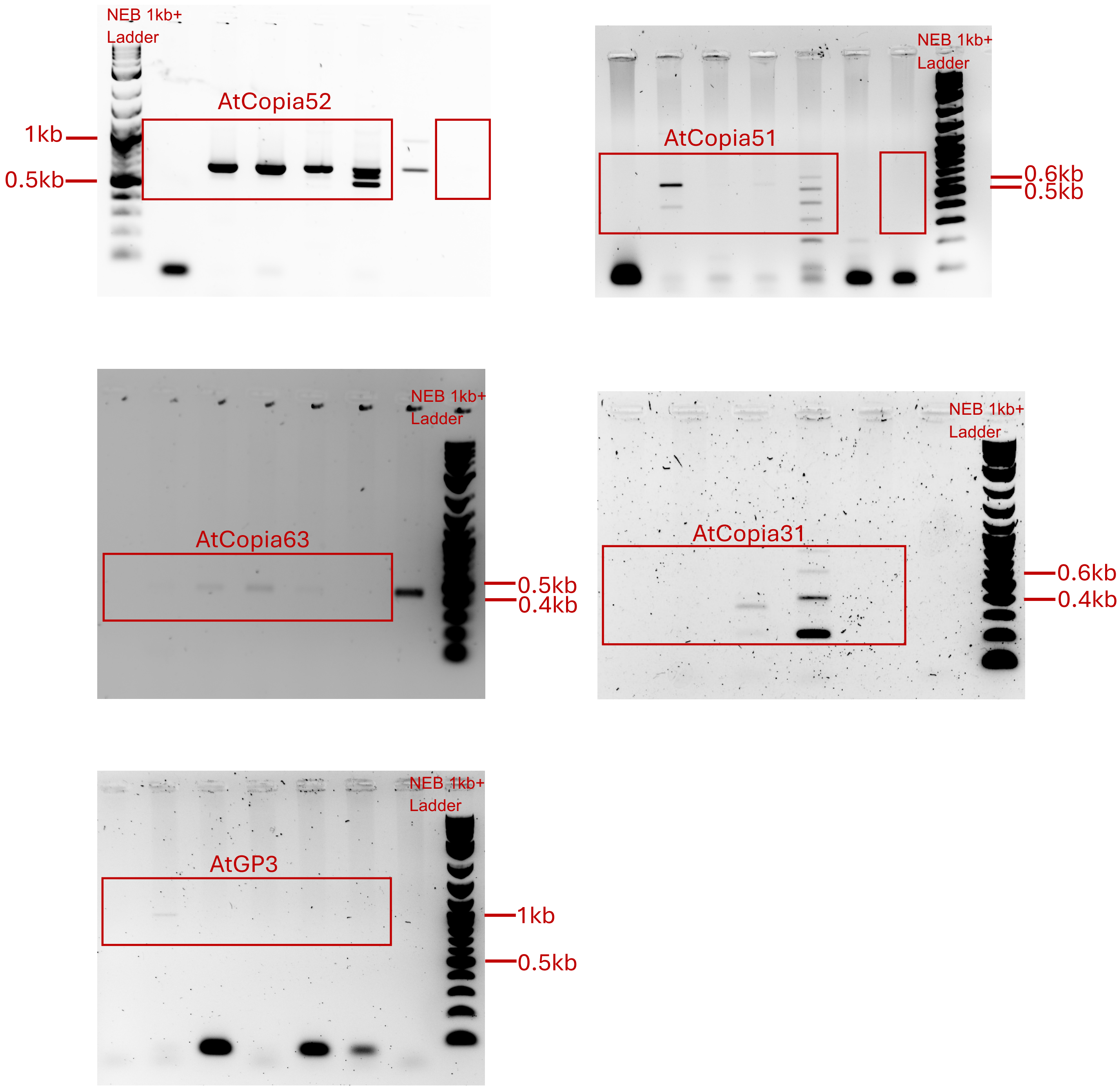

NEB 1kb+ Ladder
AtCopia52
1kb
0.5kb
AtCopia51
NEB 1kb+ Ladder
0.6kb
0.5kb
AtCopia63
NEB 1kb+ Ladder
0.5kb
0.4kb
AtCopia31
NEB 1kb+ Ladder
0.6kb
0.4kb
AtGP3
NEB 1kb+ Ladder
1kb
0.5kb

Fig. S20

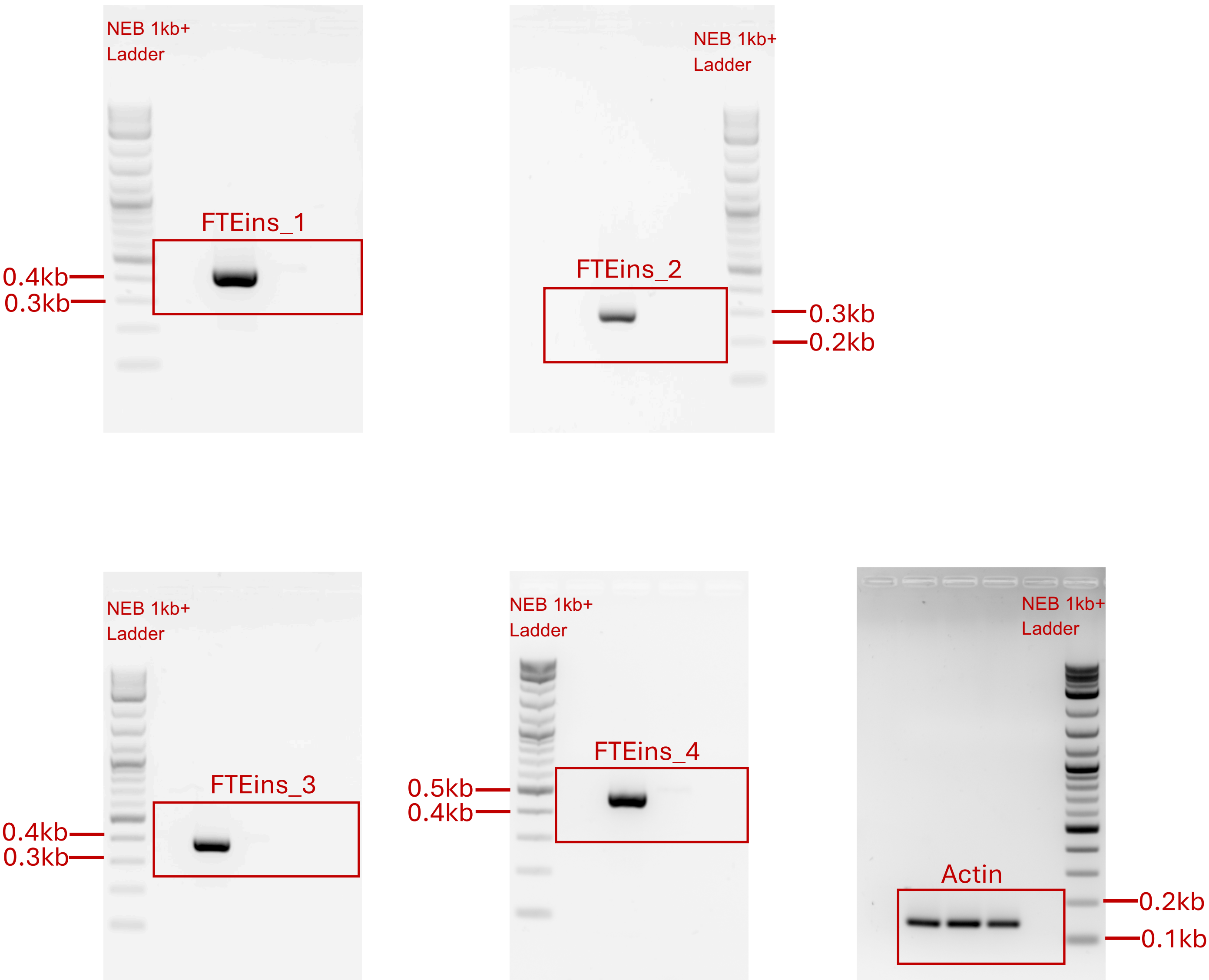
NEB 1kb+ Ladder
FTEins_1
0.4kb
0.3kb
NEB 1kb+ Ladder
FTEins_2
0.3kb
0.2kb
NEB 1kb+ Ladder
FTEins_3
0.4kb
0.3kb
NEB 1kb+ Ladder
FTEins_4
0.5kb
0.4kb
NEB 1kb+ Ladder
Actin
0.2kb
0.1kb
